# Vascular adhesion protein-1 is elevated in primary sclerosing cholangitis, is predictive of clinical outcome and facilitates recruitment of gut-tropic lymphocytes to liver in a substrate-dependent manner

**DOI:** 10.1136/gutjnl-2016-312354

**Published:** 2017-04-20

**Authors:** Palak J Trivedi, Joseph Tickle, Mette Nåmdal Vesterhus, Peter J Eddowes, Tony Bruns, Jani Vainio, Richard Parker, David Smith, Evaggelia Liaskou, Liv Wenche Thorbjørnsen, Gideon M Hirschfield, Kaisa Auvinen, Stefan G Hubscher, Marko Salmi, David H Adams, Chris J Weston

**Affiliations:** 1 National Institute of Health Research Birmingham Liver Biomedical Research Centre Institute of Immunology and Immunotherapy, University of Birmingham, Birmingham, UK; 2 Liver Unit, University Hospitals Birmingham Queen Elizabeth, Birmingham, UK; 3 Department of Transplantation Medicine, Division of Cancer Medicine, Surgery and Transplantation, Norwegian PSC Research Center, Oslo University Hospital Rikshospitalet, Oslo, Norway; 4 National Centre for Ultrasound in Gastroenterology, Haukeland University Hospital, Bergen, Norway; 5 Department of Internal Medicine IV, University Hospital Jena, Germany; 6 Center for Sepsis Control and Care, University Hospital Jena, Germany; 7 Biotie Therapies Corp., Turku, Finland; 8 MediCity Research Laboratory, University of Turku, Turku, Finland; 9 Department of Medical Microbiology and Immunology, University of Turku, Turku, Finland; 10 Department of Cellular Pathology, University Hospitals Birmingham Queen Elizabeth, Birmingham, UK

**Keywords:** IMMUNE-MEDIATED LIVER DAMAGE, PRIMARY SCLEROSING CHOLANGITIS, INFLAMMATORY BOWEL DISEASE, ULCERATIVE COLITIS, MUCOSAL IMMUNITY

## Abstract

**Objective:**

Primary sclerosing cholangitis (PSC) is the classical hepatobiliary manifestation of IBD. This clinical association is linked pathologically to the recruitment of mucosal T cells to the liver, via vascular adhesion protein (VAP)-1-dependent enzyme activity. Our aim was to examine the expression, function and enzymatic activation of the ectoenzyme VAP-1 in patients with PSC.

**Design:**

We examined VAP-1 expression in patients with PSC, correlated levels with clinical characteristics and determined the functional consequences of enzyme activation by specific enzyme substrates on hepatic endothelium.

**Results:**

The intrahepatic enzyme activity of VAP-1 was elevated in PSC versus immune-mediated disease controls and non-diseased liver (p<0.001). The adhesion of gut-tropic α4β7^+^lymphocytes to hepatic endothelial cells in vitro under flow was attenuated by 50% following administration of the VAP-1 inhibitor semicarbazide (p<0.01). Of a number of natural VAP-1 substrates tested, cysteamine—which can be secreted by inflamed colonic epithelium and gut bacteria—was the most efficient (yielded the highest enzymatic rate) and efficacious in its ability to induce expression of functional mucosal addressin cell adhesion molecule-1 on hepatic endothelium. In a prospectively evaluated patient cohort with PSC, elevated serum soluble (s)VAP-1 levels predicted poorer transplant-free survival for patients, independently (HR: 3.85, p=0.003) and additively (HR: 2.02, p=0.012) of the presence of liver cirrhosis.

**Conclusions:**

VAP-1 expression is increased in PSC, facilitates adhesion of gut-tropic lymphocytes to liver endothelium in a substrate-dependent manner, and elevated levels of its circulating form predict clinical outcome in patients.

**Significance of this study**

**What is already known on this subject?**

- Primary sclerosing cholangitis (PSC) is an immune-mediated hepatobiliary disease strongly associated with IBD, usually colitis.
- Under normal circumstances, expression of mucosal addressin cell adhesion molecule (MAdCAM)-1 is restricted to the gut, but in PSC it can also be found on hepatic endothelial cells, where it is responsible for the recruitment of mucosal effector α4β7 positive T-cells to the liver.
- We have recently shown that the aberrant expression of MAdCAM-1 in PSC liver may involve a combination of inflammation and activation of the enzyme vascular adhesion protein (VAP)-1, a potent amine oxidase.

**What are the new findings?**

- Hepatic VAP-1 enzyme activity is increased in PSC compared with non-inflamed liver, and expressed at a similar level to resected colon from patients with colitis.
- VAP-1 can catabolise a diverse range of substrates, with the most potent inducer of enzyme activity being cysteamine—an amine that can be released by the colonic epithelium and enteric pathogens.
- The degree to which VAP-1 enzyme activity facilitates the adhesion of α4β7 T-cells to hepatic endothelium is substrate-dependent.
- Circulating soluble VAP-1 levels (sVAP-1) are heightened in patients with PSC, correlate with disease severity and can be used to prospectively stratify clinical outcome.

**How might it impact on clinical practice in the foreseeable future?**

- The ability of VAP-1 to catabolise amine substrates, potentially secreted by gut epithelium or enteric pathogens, provides a theoretical link between altered colonic microbiota and mucosal immunity in PSC disease pathogenesis.
- sVAP-1 represents a biologically linked, mechanistically driven serum marker of clinical outcome in patients with PSC.
- Modulating the recruitment of mucosal lymphocytes to the liver by targeting VAP-1 highlights an avenue for therapeutic exploration.

## Introduction

Primary sclerosing cholangitis (PSC) is a progressive fibroinflammatory cholangiopathy strongly associated with colonic IBD.^1–3^ This clinical phenotype has generated several hypotheses linking inflammation in the gut and liver, one of which centres on aberrant lymphocyte homing.^4^ ^5^ Ordinarily, the gut and liver harbour distinct endothelial phenotypes, which provide a mechanism to compartmentalise tissue-specific lymphocyte recruitment.^6^ In the gut, expression of mucosal addressin cell adhesion molecule (MAdCAM)-1 on mucosal endothelium is responsible for recruiting intestinal lymphocytes that are imprinted with tissue-specific tropism; specifically those which express the MAdCAM-1 receptor, α4β7.^7^ Under normal circumstances, expression of MAdCAM-1 is restricted to the gut, but in PSC can also be detected on hepatic endothelium where it promotes recruitment of mucosal α4β7^+^T cells to the liver.^8^ The latter population are predominantly effector memory lymphocytes,^8^ ^9^ which upon reactivation result in a T cell rich portal infiltrate within the liver, and an iterative inflammatory response therein.

**Significance of this study**

- **How might it impact on clinical practice in the foreseeable future?**
- The ability of VAP-1 to catabolise amine substrates, potentially secreted by gut epithelium or enteric pathogens, provides a theoretical link between altered colonic microbiota and mucosal immunity in PSC disease pathogenesis.
- sVAP-1 represents a biologically linked, mechanistically driven serum marker of clinical outcome in patients with PSC.
- Modulating the recruitment of mucosal lymphocytes to the liver by targeting VAP-1 highlights an avenue for therapeutic exploration.

The factors leading to aberrant expression of MAdCAM-1 in PSC liver are not known, but may involve inflammation and activation of vascular adhesion protein (VAP)-1,^10^ ^11^ an adhesion molecule that is also endowed with potent amine oxidase activity. In humans, VAP-1 is constitutively expressed on the hepatic endothelial surface,^12^ ^13^ and through its catalytic function, VAP-1-mediated oxidation of methylamine can induce MAdCAM-1 expression by endothelial cells.^10^ However, VAP-1 is potentially capable of catabolising a diverse range of substrates beyond methylamine; and it is plausible that in the presence of enteric dysbiosis and/or an inflamed ‘leaky’ gut,^14^ increased portal vein amine levels lead to intrahepatic enzyme activation, upregulated hepatic endothelial MAdCAM-1 expression and recruitment of mucosal effector T cells to the liver.^4^ ^5^

The aims of this study were to compare VAP-1 expression in PSC to that observed in non-diseased liver, correlate serum levels with patient characteristics and clinical outcome, and determine the functional consequences of enzyme activation through a divergent amine substrate panel.

## Materials and methods

### Human tissue samples

Liver tissue was acquired during transplantation from patients with chronic end-stage disease, and from healthy donor livers surplus to requirements. Colonic tissue was procured via surgical resections in colitis refractory to medical treatment (UC), and distal-to-tumour segments in non-colitis associated colonic cancer (NC). All samples were obtained with Local Research and Ethics Committee approval and informed patient consent (Local Research and Ethics Committee Birmingham references: 2003/242, renewed 2012; and 06/Q2702/61).

### Demonstration and quantification of VAP-1 in human tissue

Prior to determining VAP-1 expression in human liver, representative tissue sections were stained with Oil Red O (Sigma), which stains neutral triglycerides and lipids. Steatosis was quantified by the proportion of section area that stained positive for dye as assessed by ImageJ analysis.^15^ ^16^ Sections were only used for study when 2% or less of the surface area stained positive, indicating minimal or no excess steatosis.

#### Immunohistochemistry

Chromogenic staining was used to visualise VAP-1 in frozen liver tissue and confocal microscopy to confirm cellular localisation^12^ ^17^ (see online supplementary table S1). All immunohistochemical sections were assessed by a specialist hepatopathologist.

10.1136/gutjnl-2016-312354.supp1 supplementary data

#### Absolute quantification of VAP-1 mRNA

Quantification of VAP-1 mRNA expression in human liver was assessed by qRT-PCR (see online supplementary table S2). Given the absence of a single, known housekeeping gene exhibiting stability across all aetiologies and between varying stages of human liver injury,^18^ ^19^ mRNA expression was determined by absolute quantification of samples. Briefly, copy number of VAP-1 in matched amounts of starting total RNA from each sample was measured via a calibration curve of known dilutions of a linearised plasmid encoding the *VAP-1* gene ranging from 10 copies/µL to 10^5^ copies/µL, as previously described.^12^ All starting mRNA concentrations were standardised and qRT-PCR reactions performed using 100 ng starting material run in triplicate wells.

#### VAP-1 enzyme assays

VAP-1 enzyme activity was determined in protein lysates from liver and colon using Amplex-UltraRed Reagent as a stoichiometric fluorogenic substrate for horseradish peroxidase and by quantifying the rate of H_2_O_2_ production during a deamination/oxidation reaction (see online supplementary table S3).^13^ To ensure all measured output was specific to VAP-1, test wells were always run in parallel to identical samples containing the urea derivative semicarbazide (250 µM; Sigma), which is a complete inhibitor of VAP-1 activity.^13^ The value from semicarbazide-treated samples was then subtracted from the enzymatic rate in inhibitor-free wells.

### Evaluating substrate-dependent variations in enzyme kinetics

Putative, naturally occurring substrates of human VAP-1 were selected based on inclusion in the human metabolome database V.2.5 (http://www.hmdb.ca), and with reference to Shen *et al*,^20^ specifically cysteamine, methylamine, dopamine, ethylamine and phenethylamine (Sigma). A 96-well plate was loaded with 50 ng recombinant VAP-1 (Biolegend) in 100 µL Dulbecco's phosphate-buffered saline with increasing concentrations of test substrate supplemented to test wells. A H_2_O_2_ standard curve was created, and enzyme activity quantified using the Amplex-UltraRed detection system across increasing concentrations of test substrate for a fixed concentration of VAP-1 under atmospheric conditions. Kinetic parameters were estimated by Michaelis-Menten methodology (see online supplementary figure 1). Benzylamine served as an internal positive control.

### Determining the consequences of VAP-1 activation

Hepatic endothelial cells (HEC) were extracted from whole liver and grown till confluence as previously described.^10^ ^21^

#### Cell-based ELISAs

An ELISA of cultured HEC (passage 3–4) was used to investigate the expression of MAdCAM-1, following cell stimulation with tumour necrosis factor (TNF)α (20 ng/mL; Peprotech, UK) in the absence or presence of amine substrates at concentrations yielding the maximum apparent enzymatic rate (V_max_^app^) (see online supplementary file 1).

#### Flow-based adhesion assays

Flow-based adhesion assays were conducted to assess the functional consequences of amine exposure and VAP-1 inhibition on HEC-mediated lymphocyte recruitment, as described previously.^10^ ^21^ Briefly, confluent monolayers of HEC were cultured in microcapillary flow chamber microslides (Ibidi, Germany), and stimulated with TNFα (20 ng/mL) ± amine substrate supplemented HEC media, in the absence or presence of semicarbazide (250µM) (see online supplementary file 2).

To obtain purified CD3^+^ α4β7^+^ T cells, peripheral blood lymphocytes were subject to fluorescence-activated cell sorting (MoFlo XDP High-Speed Cell Sorter in purity mode; Beckman Coulter) (see online supplementary table S4). Isolated cells were incubated overnight (Roswell Park Memorial Institute medium+10% foetal calf serum in a 48-well culture plate; Corning Costar) to facilitate recycling of surface receptors. The following day, cells were washed, pelleted and viability confirmed by Trypan blue exclusion, before being resuspended (10^6^ cells/mL) in serum-free basal endothelial media containing 0.1% bovine serum albumin, ready for perfusion.

#### Quantification of circulating soluble VAP-1 in clinical samples

Soluble (s)VAP-1 values were quantified in human circulation using a europium-coupled, time-resolved immunofluorometric assay (see online supplementary table S5), and results validated through a chemiluminescence detection technique as per Aalto *et al*^22^ (see online supplementary file 3). Serum samples were obtained from patients with PSC, primary biliary cholangitis/PBC, autoimmune hepatitis/AIH, UC alone/UC and healthy volunteers without evidence of liver disease or IBD. Notably, as liver biopsy no longer forms routine standard of care in PSC or PBC,^23^ in the absence of histological data, the presence of cirrhosis was identified according to a combination of clinical features (ascites/varices/hepatic encephalopathy), laboratory indices (hypoalbuminaemia/thrombocytopenia/prolonged international normalised ratio) and radiology (coarse or irregular liver ± reversed portal flow/splenomegaly/ascites).

#### Statistical analysis

Non-parametric data are presented using median and IQRs unless otherwise specified. The Mann-Whitney test was conducted when comparing continuous data between two independent groups, and Kruskal-Wallis test (p<0.001) with Bonferroni-Dunn post hoc correction for multiple groups. Non-parametric measures of statistical dependence between continuous variables were conducted using Spearman's rank correlation coefficient.

In prospective clinical outcome studies, time-zero was set as the point of blood sampling, and the primary endpoint defined as transplant-free survival; patients without a clinical event were censored at date of last clinic follow-up. The impact of continuous variables on clinical endpoints was evaluated by receiver operator characteristic curve analysis, and an optimal cutpoint selected (Youden Index) from a derivation cohort (Birmingham, UK) before being tested in a validation series of patients (Oslo, Norway). Cox proportional hazards models were fit to assess the impact of individual covariates on the instantaneous rate of events, with time-to-event analysis ascertained through Kaplan-Meier estimates (SPSS V.21; IBM, USA).

## Results

### VAP-1 expression is altered in PSC liver

In non-diseased liver, VAP-1 expression as shown by immunohistochemistry was largely confined to the walls of blood vessels and the hepatic sinusoids, with the latter being most evident in centrilobular regions (see online supplementary figure 2A–C). VAP-1 expression was increased in immune-mediated liver disease, including PSC, wherein sinusoidal staining was present throughout cirrhotic nodules (see online supplementary figure 2D) (see online supplementary figure 3).

We have previously shown that VAP-1 is expressed by HEC, in addition to α smooth muscle actin (αSMA) hepatic stellate cells and activated myofibroblasts in cirrhosis.^12^ To this effect, strong VAP-1 expression was seen in the fibrous septa and the walls of portal/septal vessels in PSC liver (see online supplementary figure 2E–F). Confocal immunofluorescence confirmed VAP-1 colocalisation with αSMA in the walls of portal/septal vessels and stromal cells within fibrous septa (see online supplementary figure 4). VAP-1 staining in portal/septal vessels was present in close proximity to CD31^+^ endothelial cells lining these vessels (see online supplementary figure 5), as previously described.^12^ Conversely, VAP-1 did not colocalise with EpCAM^+^ bile ducts or proliferating bile ductules in PSC liver (see online supplementary figure 6).

To quantify the level of hepatic VAP-1 expression, gene transcription was measured using qRT-PCR on cDNA derived from human liver tissue. Levels of VAP-1 mRNA were significantly greater in diseased versus non-diseased liver explants, with the highest levels seen in PSC (p=0.003) (see online supplementary figure 7).

### VAP-1 enzyme activity is increased in PSC liver

**Figure 1:**
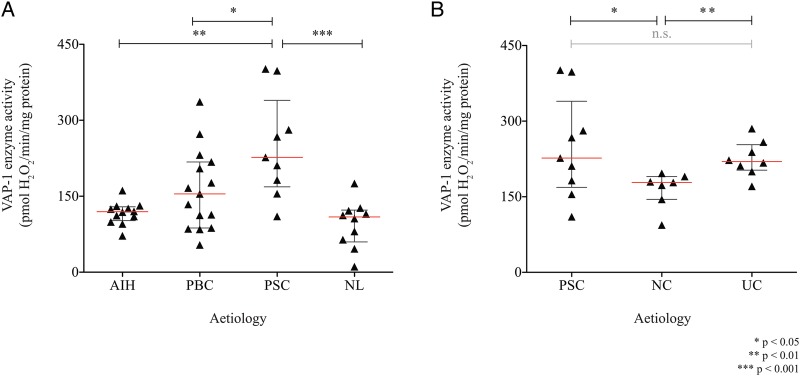
Vascular adhesion protein-1 (VAP-1) enzyme activity in human liver and colon. (A) VAP-1 enzyme activity in tissue lysates from explanted primary sclerosing cholangitis (PSC) liver (n=9) is compared with autoimmune hepatitis (AIH; n=12), primary biliary cholangitis (PBC; n=15) and non-diseased resected liver (NL; n=10); and (B) resected tissue from distal-to-tumour segments of non-colitis associated cancer (NC; n=7) and UC (n=8). Mean enzyme activity of three repeats per patient is presented.

Consistent with immunohistochemical staining patterns and qRT-PCR, we found that VAP-1 enzyme activity was elevated in PSC versus normal liver (p<0.001) (figure 1A). Given the clinical association with IBD, VAP-1 enzyme activity was also assessed in resected colonic tissue. Levels in UC were similar to those of PSC liver and greater than that in non-inflamed human colon (figure 1B).

### VAP-1 enzyme activity supports the adhesion of α4β7^+^ lymphocytes to HEC

**Figure 2:**
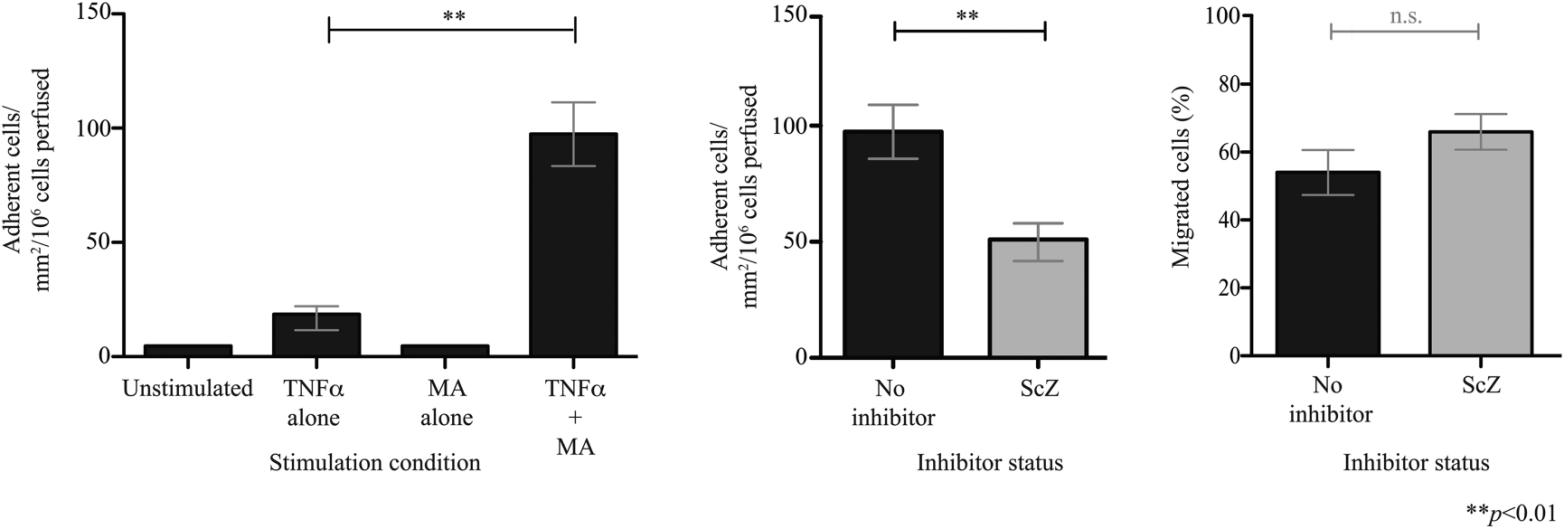
Vascular adhesion protein-1 (VAP-1) supports adhesion of α4β7^+^ lymphocytes to hepatic endothelial cells (HEC). Circulating α4β7^+^ T cells were enriched from blood donors to over 90% purity, perfused over a HEC monolayer under flow (0.05 Pa) and the number of adherent cells recorded. (A) HEC were stimulated with tumour necrosis factor (TNF)α (20 ng/mL), methylamine (MA, 100 µM) or both for 4 hours. (B) The impact of VAP-1 enzyme inhibition was tested using semicarbazide (ScZ). Data represent the mean of n=3 (±SD).

It has previously been demonstrated that VAP-1 enzyme activity enhances MAdCAM-1-dependent binding of an α4β7-expressing β-lymphoblastoid cell line to HEC.^10^ We now show that highly purified α4β7^+^ T cells isolated from peripheral blood (see online supplementary figure 8) also undergo adhesion to hepatic endothelium on which VAP-1 enzyme activity is activated by the model substrate methylamine (figure 2). This process was inhibited by approximately 50% with the selective VAP-1 enzyme inhibitor semicarbazide, although no significant differences in the proportion of cells undergoing transmigration were seen. These findings are consistent with the role of MAdCAM-1/α4β7 in the lymphocyte recruitment cascade where they support adherence but not transmigration.^10^

### VAP-1 enzymatic activity is substrate-dependent

**Figure 3:**
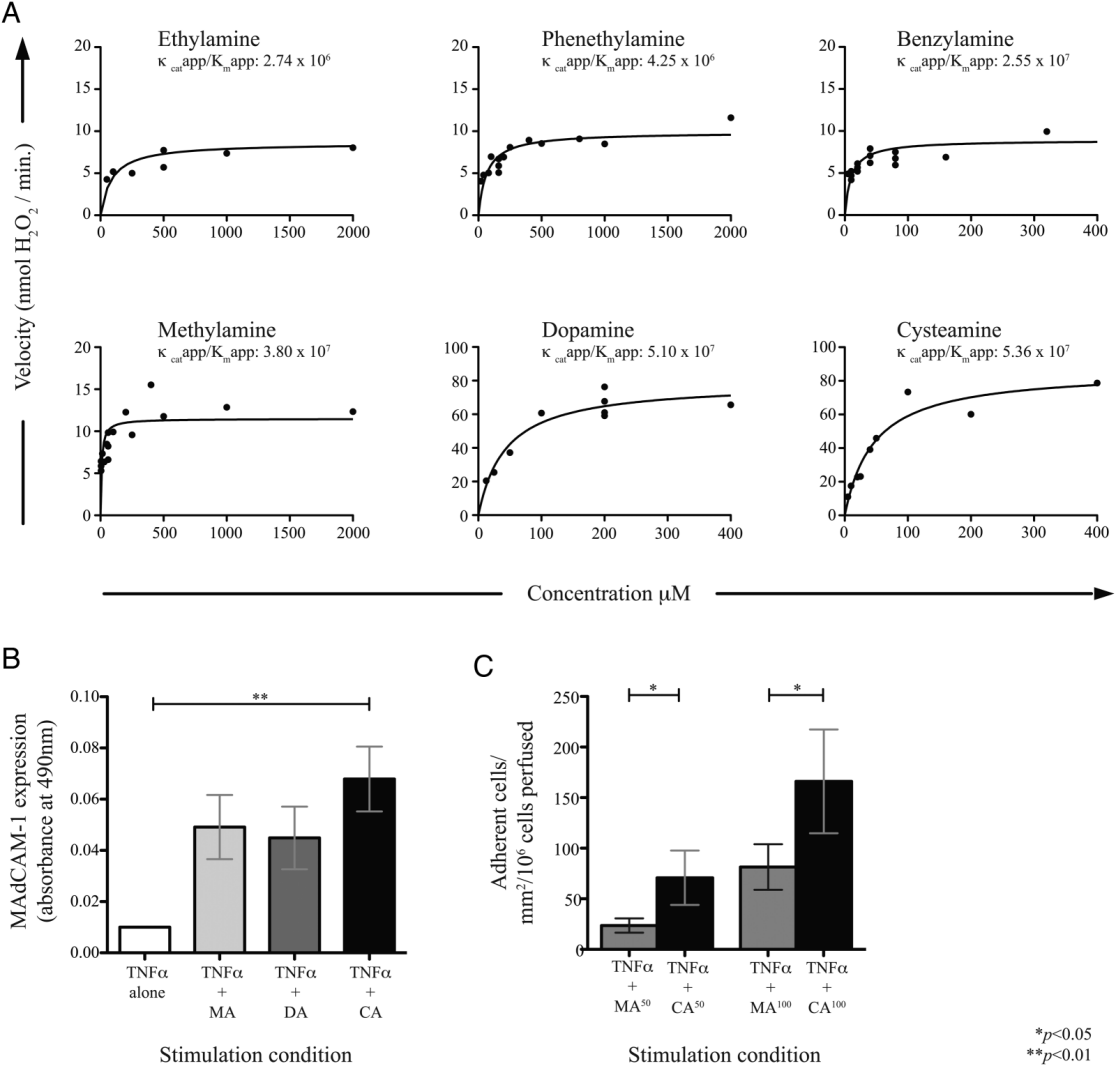
Kinetic profiling of vascular adhesion protein (VAP-1) amine substrates. (A) Non-linear fit curves showing vascular adhesion protein (VAP)-1 activity across a range of concentrations of potential substrates. Enzymatic rates are measured by Amplex-UltraRed. Each point represents mean of three replicates. (B) Mucosal addressin cell adhesion molecule (MAdCAM)-1 expression by hepatic endothelial cells (HEC) following stimulation for 4 hours with tumour necrosis factor (TNF)α alone, or plus substrates methylamine (MA), dopamine (DA) or cysteamine (CA) at concentrations yielding V_max_^app^ (n=3). (C) Flow-based adhesion assays of α4β7^+^ cells perfused across HEC treated for 4 hours with TNFα alone or in combination with MA or CA at 50–100 µM. Mean±SD of three experiments shown.

To profile the enzymatic rate following provision of other naturally occurring substrates, the amine oxidase activity of a fixed amount of purified VAP-1 was quantified across increasing substrate concentrations until the apparent maximum saturating enzyme activity was reached (V_max_^app^), and the resulting kinetic rates measured (figure 3A). Following a series of dose-finding studies and by approximating Michaelis-Menten constants, we were able to develop a hierarchy of substrates based on enzymatic efficiency (*k*_cat_^app^/*K*_M_^app^) with the highest rate seen for cysteamine (see online supplementary table S6). This was of particular interest given that cysteamine can be generated by the colonic epithelium and induce colitis,^24–30^ and also by bacteria associated with enteric dysbiosis in PSC-IBD.^14^

A cell-based ELISA was then used to measure the impact on MAdCAM-1 induction in response to varying substrate provision (figure 3B). Again, cysteamine was shown to be the most potent of all amines tested, resulting in significantly higher MAdCAM-1 expression by HEC than methylamine and dopamine (another potent VAP-1 substrate). All experiments were carried out in the presence of TNFα to mimic local inflammation.^21^ ^31–34^ Moreover, cysteamine-stimulated HEC supported adhesion of α4β7^+^ T cells under flow to a significantly greater degree than that observed with methylamine at matched concentrations (figure 3C), suggesting that as a substrate, cysteamine is also associated with greatest VAP-1 efficacy.

### Circulating soluble VAP-1 levels are elevated in the sera of patients with PSC

**Figure 4:**
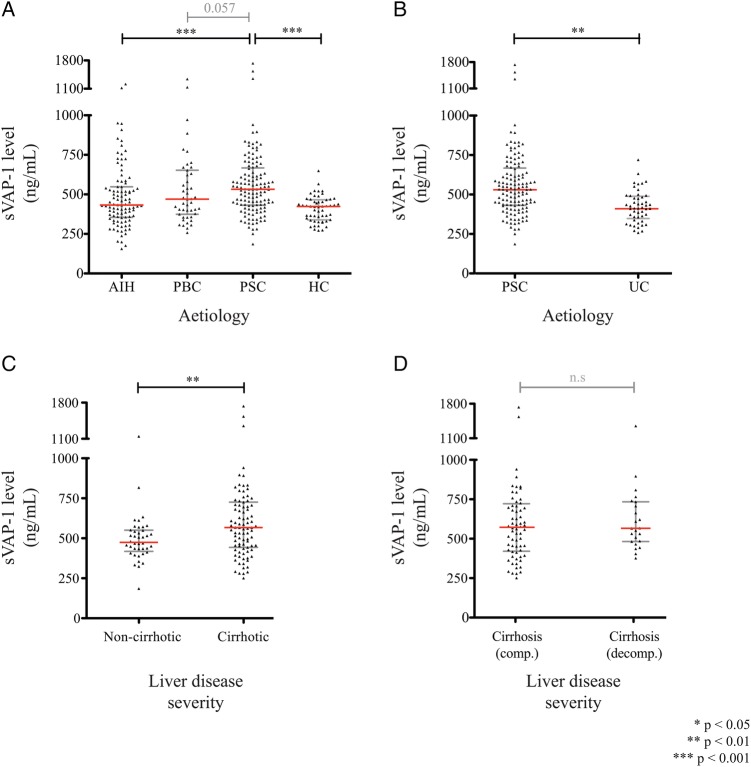
Correlation of serum vascular adhesion protein-1 (sVAP-1) levels with disease severity in primary sclerosing cholangitis (PSC). (A) Circulating soluble vascular adhesion protein-1 (sVAP-1) levels measured via immunofluorescence in PSC (n=134) versus autoimmune hepatitis (AIH, n=90), primary biliary cholangitis (PBC, n=48) and healthy controls (HC, n=54); and (B) versus UC without evidence of PSC (n=50). (C) Serum sVAP-1 levels in patients with PSC are shown according to the severity of liver disease: non-cirrhotic (n=39) versus established cirrhosis (n=95); and (D) in cirrhotic patients with compensated (n=68) versus decompensated liver disease (n=27).

In addition to its expression within the liver, VAP-1 can be released into the circulation in soluble form (sVAP-1).^35^ The hepatic vasculature is an important source of sVAP-1, and we have recently shown that it is also released by activated hepatic stellate cells and liver myofibroblasts—key cellular mediators of tissue fibrosis.^12^ We measured serum sVAP-1 in a cohort of patients under active follow-up using an immunofluorescence ELISA (table 1), and found higher values in those with PSC (n=134; 532 ng/mL, IQR 432–668 ng/mL) compared with AIH (n=97; 433 ng/mL, IQR 355–548 ng/mL), PBC (n=48; 470 ng/mL, IQR 374–653 ng/mL), healthy control subjects (n=54; 425 ng/mL, IQR 336–466 ng/mL) and patients with colitis alone (n=50; 413 ng/mL, IQR 348–489 ng/mL) (figure 4A,B).

**Table 1:** Demographic variation across sample populations

	PSC (n=134 overall)*	PBC (n=48)	AIH (n=97)	IBD (n=50)	HC (n=54)
Patient age†	42 (29–59) yrs.	55 (49–63)	49 (29–61)	35 (26–54)	34 (38–37)
Male gender	83 (62)	3 (6)	21 (21)	31 (62)	43 (80)
UDCA exposure within past 3 months	96 (72)	34 (71)	1 (1)		
Laboratory parameters†					
Serum AST	66 (34–100) IU/L	40 (26–80) IU/L	31 (23–48) IU/L		
Serum ALT	61 (34–105) IU/L	35 (25–77 IU/L	26 (20–52) IU/L		
Serum ALP (ratio to ULN)‡	2.50 (1.80–4.11)	2.39 (1.43–3.34)	1.25 (1.11–1.81)		
Bilirubin	20 (11–48) µmol/L	13 (7–24) µmol/L	10 (7–14) µmol/L		
Albumin	43 (38–45) g/dL	42 (40–45) g/dL	44 (41–47) g/dL		
Platelet count	200 (128–298)×10^3^ cells/mm^3^	247 (153–310)×10^3^ cells/mm^3^	221 (139–283)×10^3^ cells/mm^3^		
INR	1.1 (1.0–1.2)	1.0 (0.9–1.1)	1.0 (1.0–1.1)		
Sodium	141 (139–142) mmol/L	141 (140–143) mmol/L	142 (140–144) mmol/L		
Creatinine	72 (58–82) µmol/L	64 (58–71) µmol/L	66 (60–75) µmol/L		
IgG	14.17 (11.77–17.71) g/L	14.59 (11.18–17.74) g/L	14.16 (10.68–16.97) g/L		
ANA-positive	60 (45)	21 (49)	68 (70)		
ASMA-positive	41 (31)	11 (26)	64 (66)		
Cirrhosis†	95 (71)	24 (50)	45 (46)		
Decompensated	28	6	4		
MELD score†	6 (6–10)	6 (6–7)	6 (6–6)		
Child-Turcotte-Pugh score†					
A	78 (58)	35 (73)	86 (87)		
B	52 (39)	10 (21)	9 (9)		
C	4 (3)	1 (2)	2 (2)		
Clinical events	56	16	4		
Liver transplantation	44	11	0		
Death	12	5	4		

Data for categorical variables expressed as number with percentages in parenthesis. Continuous variables expressed as median (IQR).

*Three patients with small-duct PSC.

†At time of sampling.

‡Ratio to upper limit of normal provided, given variation between assay methods over time.

AIH, autoimmune hepatitis; ALP, alkaline phosphatase; ALT, alanine transaminase; ANA, antinuclear antibody; ASMA, anti-smooth muscle antibody; AST, aspartate transaminase; HC, healthy controls; IgG, immunoglobulin G; INR, international normalised ratio; MELD, model for end-stage liver disease score, PBC, primary biliary cholangitis; PSC, primary sclerosing cholangitis; UDCA, ursodeoxycholic acid; ULN, upper limit of normal.

A greater number of patients with PSC displayed features of established cirrhosis, together with an increased number of clinical events over time compared with our PBC and AIH groups. In light of this observation, we then stratified our autoimmune liver disease cohort according to elevated sVAP-1 values, via dichotomisation at the median for the overall cohort (cut-off: 485 ng/mL). To this effect, PSC retained a significant association with heightened sVAP-1 independently of the presence of liver cirrhosis (adjusted OR 1.86, 95% CI 1.10 to 3.14; p**=**0.021).

### sVAP-1 is associated with disease severity and predictive of clinical outcome

When patients with PSC were studied in more detail, elevated values were seen particularly in those with cirrhosis compared with non-cirrhotic individuals, but with no statistically significant differences between compensated versus decompensated disease (figure 4C,D) (table 2).

**Table 2:** Soluble VAP-1 levels according to clinical covariates

Categorical associations	sVAP-1 level (ng/mL)			p Value
Sex				
Male vs female	529 (432–627)		567 (457–698)	0.325
Disease severity				
Non-cirrhotic vs Cirrhotic	462 (406–547)		578 (413–736)	0.006
Ascending cholangitis within past 3 months*				
Presence vs absence	550 (376–646)		532 (438–672)	0.350
IBD†				
Presence vs absence	532 (431–653)		542 (438–663)	0.668
Active vs inactive	523 (387–626)		533 (443–672)	0.466
Colectomy vs colon intact	534 (443–710)		532 (434–651)	0.939
Immunosuppression within past 3 months				
Exposure vs absence	516 (418–621)		549 (438–663)	0.630
UDCA exposure within past 3 months				
Treatment vs non-treatment	534 (451–681)		457 (413–571)	0.053
Antibiotic exposure within past 3 months‡				
Exposure vs absence	566 (395–621)		529 (436–672)	0.600
ANA				
Positive vs negative	517 (432–696)		551 (435–631)	0.857
ASMA				
Positive vs negative	508 (421–627)		543 (436–676)	0.462
pANCA				
Positive vs negative	517 (436–667)		552 (436–663)	0.794
Child-Turcotte-Pugh score				
A vs B vs C	512 (406–598)	559 (453–750)	524 (430–699)	0.347
**Continuous variable correlations**	**Spearman's ρ**			**p Value**
Patient age	0.194			0.027
Serum AST	−0.002			0.993
Serum ALT	−0.076			0.691
Serum ALP (ratio to ULN)	−0.205			0.278
Serum bilirubin	0.217			0.250
Serum albumin	−0.819			0.318
INR	0.014			0.941
Platelet count	−0.585			0.001
Serum sodium	0.141			0.113
Serum creatinine	−0.242			0.197
MELD score	0.128			0.149
IgG	0.195			0.303

Continuous data presented as medians (IQR in parenthesis).

*A total of 25 patients were noted to have an attack of ascending cholangitis within 3 months of serum sampling.

†A total of106 patients had a history of IBD (all colitis), of whom 27 had active disease. Another 19 patients had undergone prior colectomy (9 with colonic dysplasia), of which 5 were experiencing pouchitis.

‡Thirty-six patients were exposed to antibiotics within 3 months of serum sampling.

ALP, alkaline phosphatase; ALT, alanine transaminase; ANA, antinuclear antibody; ASMA, anti-smooth muscle antibody; AST, aspartate transaminase; IgG, immunoglobulin G; INR, international normalised ratio; MELD, model for end-stage liver diseases; pANCA, perinuclear antineutrophil cytoplasmic antibody; UDCA, ursodeoxycholic acid; ULN, upper limit of normal; VAP, vascular adhesion protein.

**Figure 5:**
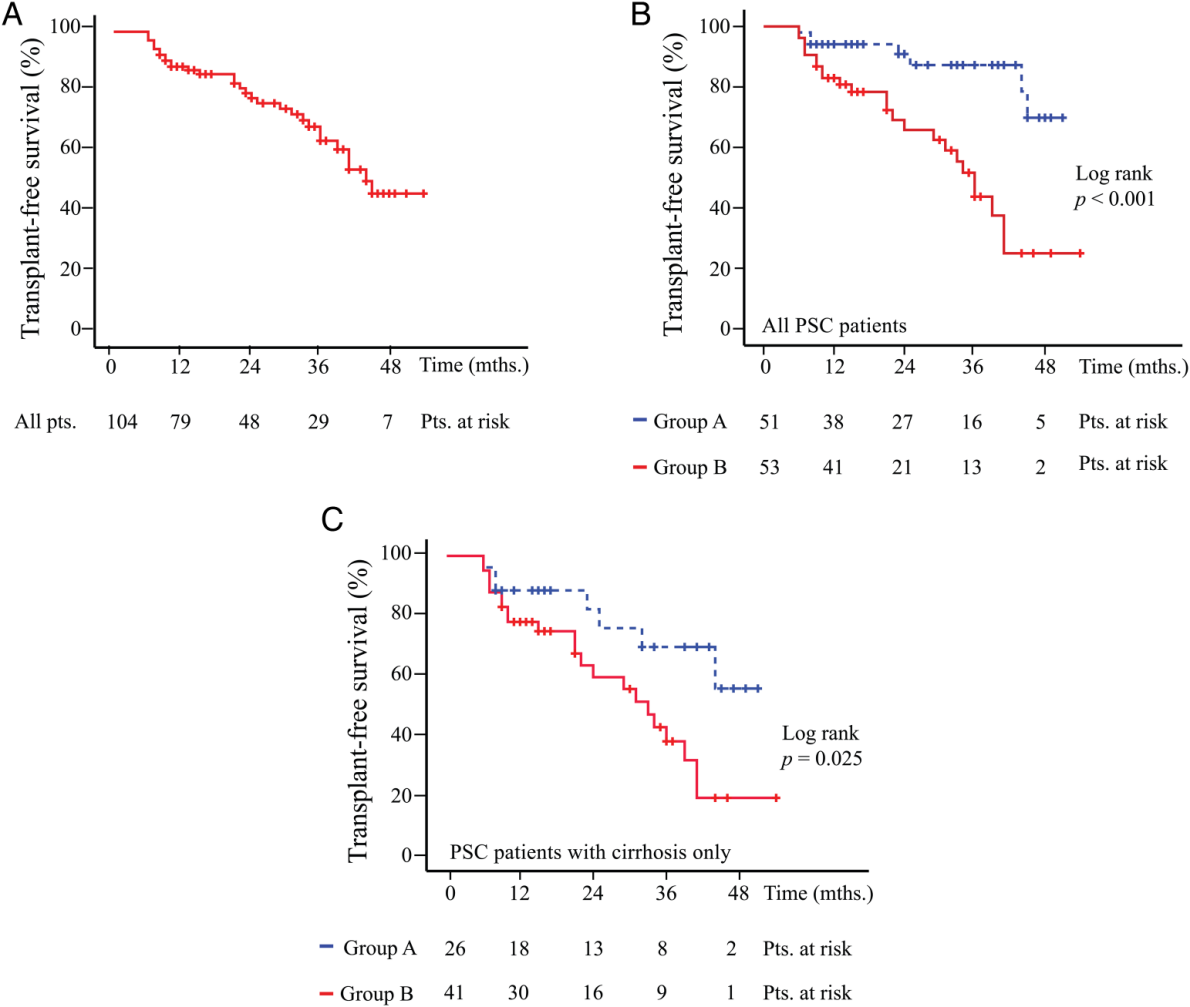
Serum vascular adhesion protein-1 (sVAP-1) values predict clinical outcome in primary sclerosing cholangitis (PSC). (A) Event-free survival from time of serum sampling, overall (prospectively evaluated; Birmingham PSC cohort); and (B) stratified according to circulating soluble (s)VAP-1 levels measured via immunofluorescence, with an area under the receiver operator characteristic curve derived cutpoint of 529 ng/mL. Group A: sVAP-1<529 ng/mL and Group B: sVAP-1>529 ng/mL. (C) A subanalysis of clinical outcome exclusively in patients with established cirrhosis using the same cut-off points. Clinical events: liver transplantation or liver-related death.

Having identified an association with indices of advanced disease, the ability of sVAP-1 to predict clinically significant endpoints was evaluated prospectively. In the Birmingham derivation cohort, 51 patients underwent liver transplantation or sustained a liver-related death (n**=**44 and n**=**7, respectively), yielding an incidence rate of 19.7 clinical events per 100 patient-years. Survival analysis was restricted to those patients not listed for liver transplantation at time of sampling and with a minimum 3-month follow-up (n=104; median event-free survival from time of serum sampling: 39.0 months, IQR 32.1–45.9 months, figure 5A), in which the prognostic value of sVAP-1 was evident on a continuous scale (see online supplementary figure 9A). An optimal cutpoint of 529 ng/mL was determined via the Youden Index method and shown to stratify clinical outcome (HR: 3.76, 1.68–8.47, p**=**0.001) (figure 5B) independent of other risk predictors on multivariable analysis (adjusted HR: 3.85, 1.68–8.47, p=0.003) (table 3).

**Table 3:** Covariates associated with future risk of death or liver transplantation in the PSC derivation cohort

	Univariate analysis		Multivariable analysis (adjusted for platelet count and serum albumin)*		Multivariable analysis (adjusted for cirrhosis)*	
	Unadjusted HR (95% CI)	p Value	Adjusted HR (95% CI)	p Value	Adjusted HR (95% CI)	p Value
Elevated serum AST (per IU/L increase)†	1.01 (1.00 to 1.01)	0.006	1.01 (1.00 to 1.02)	0.003	1.02 (1.01 to 1.02)	<0.001
Ascending cholangitis	2.62 (1.20 to 5.72)	0.016	3.40 (1.46 to 7.90)	0.004	4.23 (1.72 to 10.42)	0.002
Antibiotic exposure	1.91 (1.24 to 2.92)	0.003	NS	NS	NS	NS
Cirrhosis	6.02 (1.83 to 20.00)	0.003	N/A	N/A	7.69 (2.04 to 27.03)	0.002
Elevated serum bilirubin (per g/L increase)†	1.02 (1.012 to 1.03)	<0.001	NS	NS	N/A	
Thrombocytopenia (per 50×10^3^ cells decrease)†	1.22 (1.02 to 1.42)	0.028	NS	NS	N/A	
Hypoalbuminaemia (per g/L decrease)†	1.21 (1.12 to 1.30)	<0.001	1.18 (1.08 to 1.28)	<0.001	N/A	
sVAP-1 (per 100 ng increase)†	1.11 (1.11 to 1.22)	0.002	(Not included)		(Not included)	
sVAP-1: >529 ng/mL	3.76 (1.68 to 8.47)	0.001	2.70 (1.03 to 714)	0.043	3.85 (1.57 to 9.34)	0.003

*As platelet count and hypoalbuminaemia were used in defining cirrhosis as a covariate, their impact was determined on a singular as well as collective level in a stepwise multivariable Cox regression model.

†Values in parenthesis indicate that the degree of risk (HR in the adjacent columns) increases according to a unit change in the given variable.

**A**ST, aspartate transaminase; NA, not applicable; NS, not significant; PSC, primary sclerosing cholangitis; VAP, vascular adhesion protein.

Given that liver cirrhosis and sVAP-1 levels exhibited a significant statistical interaction (p<0.01), the additive predictive value of the latter was then assessed exclusively in a subset of patients with PSC who already had advanced liver disease. Despite this restriction, elevated sVAP-1 levels were still able to stratify the subgroup of cirrhotic patients with PSC at highest risk of liver transplantation/death (HR: 2.02, 1.17–3.51, p=0.012) (figure 5C). Reciprocally, elevated sVAP-1 values discriminated the risk of poorer clinical outcome in patient groups having a normal serum bilirubin, a normal serum albumin and/or a normal platelet count (see online supplementary figure 10).

### Validation of sVAP-1 as a biomarker for patients with PSC

To substantiate the findings of clinical outcome studies, we quantified sVAP-1 levels using a second, independent detection system (chemiluminescence), and showed strong correlation between both techniques (see online supplementary figure 11). Moreover, when applying the chemiluminescence assay, serum sVAP-1 values retained predictive value of transplant-free survival in our derivation cohort (see online supplementary figure 9B), with an optimum cutpoint of 923 ng/mL. Patients with a chemiluminescence-derived sVAP-1 concentration above this value carried a significantly increased risk of future clinical events (HR: 5.10, 1.95–13.34, p=0.001).

Next, we obtained serum samples from an external PSC cohort from Oslo (Norway), in an attempt to validate the biomarker potential of sVAP-1. Patient characteristics and comparison against the Birmingham derivation cohort are provided in online supplementary table S7. As such, elevated serum sVAP-1 in the validation cohort also associated with significantly poorer transplant-free survival (see online supplementary figure 12), characterising a group at high risk of disease progression (HR: 3.06, 1.39–6.78, p=0.006).

## Discussion

The results of our study indicate that in PSC, expression and enzyme activity of VAP-1 are increased in the liver, which is reflected by increased circulating soluble levels. These findings have important implications for understanding disease pathogenesis, because we subsequently show how activation of VAP-1 on hepatic endothelium increases the ability to recruit α4β7^+^ T cells from blood that were originally activated in the gut. Because pathophysiological substrates for VAP-1 are not known, we also studied a range of natural amines in their ability to induce enzyme activation. In so doing, we found that the most potent substrate is cysteamine which has been shown to induce colitis and colonic cancer in mice;^27–30^ an intestinal phenotype mirrored clinically in patients with PSC.^1–3^ Furthermore, cysteamine when administered to hepatic endothelium resulted in increased MAdCAM-1 expression and an enhanced ability to recruit α4β7^+^ cells under flow. This lymphocytic phenotype defines a mucosa-activated, effector memory cell population, which are known to be recruited to PSC liver consequent to aberrant endothelial expression of MAdCAM-1.^8^

Whether amine substrates are generated locally or delivered from the gut via portal circulation can only be speculated. Unfortunately, measuring amine levels in human tissue or serum is hampered by rapid amine oxidation, amine volatility and prolonged derivatisation times that preclude analysis in the clinical setting.^36^ ^37^ Nevertheless we have recently shown how mucosa-adherent microbiota in PSC are distinct, with an enrichment of the genus *Escherichia*.^14^ The latter are known to produce cysteamine in vitro, as can the potentially oncogenic genus *Enterobacter*.^20^ ^38–40^ Cysteamine may also derive directly from the intestinal epithelium, through the activity of the ectoenzyme vanin-1.^41^ Vanin-1 mediates cysteamine release in the gut, and the introduction of cysteamine promotes colonic inflammation and carcinogenesis in vivo.^28^ ^29^ ^41^ These observations have direct relevance to the increased colonic cancer risk in human PSC. Moreover, the aldehyde by-product of cysteamine deamination is 3-mercaptopropionaldehyde, which is associated with abnormal collagen crosslinking and a potential contributor to fibrogenesis in chronic liver disease.^42^ However, there are no reports of cysteamine being generated within human liver itself.

Although hepatic VAP-1 expression was increased in all immune-mediated liver diseases, this was marked in PSC and mirrored by heightened amine oxidase activity. Notably, hepatic enzyme activity in PSC was similar to that seen in the colon in IBD, and it is plausible that upregulated colonic and hepatic VAP-1 expression may have evolved to cope with an increased burden of amines generated during intestinal inflammation. In such a model, colonic dysbiosis could activate mucosal immune responses leading to colitis, and an increase in amine release from both bacteria and the inflamed epithelium. These substrates when released into the portal vein would enter the liver and activate VAP-1, modulating the expression of adhesion molecules on the hepatic endothelium.^5^ Thus, our data put forward a novel pathway through which colonic inflammation and amine release may lead to liver damage, providing further links between IBD and PSC. In support of this paradigm, mucosa-adherent bacteria in PSC have been shown to be distinct from those found in patients with IBD alone and normal, healthy colon.^14^ ^43–46^

Taken together with our previously published data, we hypothesise that VAP-1 expression is critical during multiple stages of PSC pathogenesis. In early disease, VAP-1 expression would be increased on hepatic vessels in an effort to catabolise the increased burden of amine substrates delivered via the portal circulation from an inflamed gut. The resultant increase in amine oxidase activity would drive upregulation of endothelial adhesion molecules (including MAdCAM-1), fostering recruitment of α4β7^+^ mucosal lymphocytes and leading to a proinflammatory response within the liver. Additionally, the increased expression we observe in fibrous septa and αSMA-positive cells suggest a broader role in advanced liver disease that may not be aetiology-specific, rather characterising a more generalised process contributing to tissue fibrogenesis.^12^

We also found that circulating sVAP-1 concentrations in patients with PSC were elevated versus those with IBD alone and healthy control subjects, and levels were also heightened when compared with patients with PBC and AIH. However, the variation between patients with autoimmune liver disease may reflect a more advanced stage of liver injury in our PSC cohort rather than a true aetiology-specific difference. Indeed, a large proportion of patients with PSC had evidence of cirrhosis at time of serum sampling, with a greater clinical event rate observed during follow-up compared with other immune-mediated liver diseases. While we used multivariable statistical analysis to adjust for the presence of liver cirrhosis, it would be incorrect to presume that heightened sVAP-1 levels are a feature unique to PSC, and similar caveats apply to other proposed biomarkers.^47^ ^48^ Notably, sVAP-1 levels were highest in the cirrhotic PSC subgroup and correlated with certain indices of liver disease severity. This observation is important, for elevated sVAP-1 has also been described in patients with non-alcoholic fatty liver disease, predominantly those with a more advanced stage of fibrosis,^12^ although prognostic utility in a dedicated clinical outcomes' analysis was not evident in this cohort.

Serum bilirubin and albumin are recognised clinical correlates of liver cirrhosis, and their predictive value in forecasting clinical outcomes in PSC is well described.^49^ These findings were validated in the present study, in addition to recurrent ascending cholangitis, elevated serum aspartate transaminase (AST) and the presence of liver cirrhosis being identified as further prognostic factors. Moreover, we found that circulating sVAP-1 levels independently predicted transplant-free survival in a prospective series of patients even when restricting analysis to those with cirrhosis, and reciprocally in individuals with a serum bilirubin, serum albumin and/or circulating platelet count within the normal range. This observation suggests that sVAP-1, while not aetiology-specific per se, represents a biologically driven and mechanistically linked surrogate of clinical outcome that can be applied to patients with PSC with an already advanced liver disease stage, in addition to those harbouring normal synthetic function.

Serum sVAP-1 concentrations were determined using two separate techniques applying different internal protein standards. Although the values we obtain show strong intrasample correlation between methods, levels were generally greater when assayed via chemiluminescence. Such variation between techniques is well recognised in clinical practice,^50–53^ and future work exploring the predictive utility of sVAP-1 cut-offs in PSC and other chronic liver diseases must be mindful of the analytical method applied. As with any newly proposed biomarker, sVAP-1 also requires validation across larger cohorts, with internationally agreed protein standard preparation and reference ranges set at population level. Moreover, to truly interrogate aetiology-specific differences in VAP-1 expression and soluble circulating levels, prospective large-scale collaboration is necessary, with enough statistical power to facilitate propensity score-matched analysis specifically for disease severity.^54^ Equally, it is important for future studies to establish when in the clinical course hepatic VAP-1 expression becomes increased, and the point at which circulating serum levels discriminate high-risk versus low-risk patient groups. Our study carries a further limitation in this regard, given that experiments with liver tissue were restricted to organs retrieved at transplantation, hence already afflicted with end-stage cirrhosis. However, as liver biopsy is not part of routine clinical care in PSC, access to fresh/frozen tissue from early stage/non-cirrhotic patients (although desirable) is rarely feasible for research purposes. Finally, the proposed hypothesis wherein hepatobiliary disease is driven by enteric dysbiosis and dysregulated mucosal immune responses is restricted by the fact that not all patients with PSC manifest clinically overt colitis; and conversely, not all individuals with intestinal inflammation go on to develop liver injury.

In summary, we demonstrate that hepatic VAP-1 enzymatic activity is increased in PSC, capable of supporting the recruitment of mucosal α4β7^+^ lymphocytes to the liver, and determined by potentially gut-derived substrates such as cysteamine. Moreover, serum VAP-1 values correlate with disease severity and are predictive of outcome in PSC, providing utility for risk stratification in clinical practice.^55^ Given its role in mediating α4β7/MAdCAM-1 interactions, VAP-1 may also represent a future therapeutic target, which would modulate the trafficking of lymphocytes from the inflamed gut to the liver, and potentially impact fibrogenesis.^4^ ^5^ ^12^ These pleiotropic mechanisms may explain how targeting VAP-1 carries additional benefit compared with α4 inhibition seen in murine models,^56^ and also over other strategies affecting lymphocyte recruitment more broadly, though with deleterious side effects.^57^
