# Supplementary material for: Vascular adhesion protein-1 is elevated in primary sclerosing cholangitis, is predictive of clinical outcome and facilitates recruitment of gut-tropic lymphocytes to liver in a substrate-dependent manner
Source: Gut. 2017 Apr 20;67(6):1135–45. doi: 10.1136/gutjnl-2016-312354 (PMC5969351; doi:10.1136/gutjnl-2016-312354)

Supplementary File 1: Methodology for cell-based enzyme-linked immunosorbent assays

An enzyme-linked immunosorbent assay (ELISA) of cultured HEC (passage 3–4) was used to investigate the expression of MAdCAM-1 following cell stimulation. Confluent monolayers of HEC were stimulated for four hours in a 48-well flat-bottomed plate with TNFα (20ng/mL; Peprotech, UK) in the absence or presence of amine substrates at concentrations yielding the maximum apparent enzymatic rate (V_{max}^{app}). The four-hour mark was chosen, as it was the time point following which MAdCAM-1 expression peaked (data not shown). Subsequently, HEC were washed, then fixed in 100% ice-cold methanol (Thermo Fisher) and incubated with 2% normal goat serum (Sigma) to reduce non-specific antibody binding. The wells were washed (PBS+0.1% BSA), following which primary antibody or isotype-matched control (IMC) were incubated with the cells (mouse anti-MAdCAM-1 IgG1 5 μg/mL, AbD Serotec isotype clone 314G8; mouse-IMC IgG1 5 μg/mL, R&D systems isotype clone 11711). After further washes, cells were incubated with a relevant secondary antibody conjugated to HRP (1/5000 dilution of goat anti-mouse HRP secondary IgG polyclonal, Dako), before development of the peroxidase enzyme conjugate using an OPD-based substrate in accordance with manufacturer's instructions (1,2-phenylenediamine dihydrochloride in tablet form; Dako, USA). Colour development was allowed to continue for up to 10 minutes before being stopped (0.5M sulphuric acid; Thermo Fisher). Absorbance at 490nm was measured on an automated plate reader (BioTek). Background absorbance was corrected by subtracting absorbance values of an IMC antibody and mean absorbance values calculated from readings of three technical repeats.

Supplementary File 2: Methodology for flow-based adhesion assays

Flow-based adhesion assays were conducted to assess the functional consequences of amine exposure and VAP-1 inhibition on HEC-mediated lymphocyte recruitment, as described previously [10,21]. Briefly, confluent monolayers of HEC were cultured in micro-capillary flow-chamber microslides (Ibidi, Germany), and stimulated with TNFα (20ng/mL) +/– amine substrate supplemented HEC media for four hours in the absence or presence of semicarbazide (250μM). To minimise the effect of exogenous VAP-1 present in laboratory serum, low serum endothelial media (2% FBS; no HGF/VEGF) was used during this step. Thereafter, HEC were washed and cell populations of interest perfused (wall shear stress of 0.05Pa). Adherent cells were visualised by phase-contrast microscopy (x10 objective) and classified as firmly adherent or migrated. Adherent cells are expressed as cells/$mm^2/10^6$ cells perfused, and migrated cells a proportion thereof.

Supplementary File 3: Chemiluminescence detection of serum sVAP-1 levels

White Nunc MaxiSorp® 96-well plates were coated with an anti-VAP-1 monoclonal antibody overnight (TK8-18; 10 µg/mL, overnight) and blocked with 1% gelatin and 1% non-fat milk powder, before adding serum (diluted 1:100 in blocking solution, 1 hour incubation). Thereafter, a biotinylated anti-VAP-1 monoclonal antibody (TK8-14 (5 µg/mL, in blocking solution), streptavidin-horseradish peroxidase conjugate (1:500, GE healthcare), and BM Chemiluminescence ELISA substrate (Roche) were sequentially added to the wells. Chemiluminescence signals were analyzed using a Tecan Infinite M200 plate reader (Tecan, Switzerland). Linear standard curves were generated using seven different concentrations of recombinant human VAP-1 (Peprotech) diluted in blocking solution containing VAP-1-depleted human serum. All samples were analyzed twice (on two different days), and sVAP-1 concentration counted as the mean of these two independent determinations. Control sera with low and high sVAP-1 levels were included in each ELISA plate. The inter-assay and intra-assay coefficients of variability were 4.0 % and 3.0 %, respectively.

Supplementary Table 1: Primary antibodies for immunohistochemistry

**A) Chromogenic staining**			
**Antibody (supplier)**	**Isotype (clone)**	**Stock conc.**	**Working dilution**
Mouse anti-VAP-1 (BioTie)	IgG2a (TK8-14)	1 mg/mL	1/500
Mouse anti-VAP-1 (Hycult Biotech)	IgG1 (174-5)	0.1 mg/mL	1/200
Mouse anti-EpCAM (Progen)	IgG1 (HEA125)	0.05 mg/mL	1/10
Mouse anti-CD31 (eBioscience)	IgG1 (WM59)	0.5 mg/mL	1/400
Mouse anti-MAdCAM-1 (AbD Serotec)	IgG1 (314G8)	1 mg/mL	1/500
Mouse anti-αSMA (Sigma)	IgG2a (1A4)	2 mg/mL	1/400
**IMC**			
Mouse (Dako)	IgG1 (DAK-GO1)	0.1 mg/mL	*Assay dependent*
Mouse (Dako)	IgG2a (DAK-GO5)	0.1 mg/mL	*Assay dependent*
Goat (R&D systems)	Polyclonal (Cat. # AB-108-C)	1 mg/mL	*Assay dependent*
**B) Confocal immunofluorescence**			
Goat anti-mouse AF488 (Thermo Fisher)	IgG1	100 μg/mL	1/500
Goat anti-mouse AF546 (Thermo Fisher)	IgG2a	100 μg/mL	1/500

Abbreviations: EpCAM, epithelial cell adhesion molecule; IMC, isotype-matched control; MAdCAM, mucosal addressin cell adhesion molecule; SMA, smooth muscle actin; VAP, vascular adhesion protein.

Supplementary Table 2: Reagents used for VAP-1 qRT-PCR *

**Target**	**Primer/Probe assay ID**	**Source**
VAP-1	Hs00186647_m1	Life Technologies
**Reaction mixture and consumables**		
**Reagent**	**Volume (ml)**	**Source**
TaqMan Master mix	10	Life Technologies
Probe/primer	1	Life Technologies
cDNA (synthesised from 50 µg/mL RNA)	2	N/A
Nuclease-free water	7	QIAGEN
LightCycler® 480 Multiwell Plate 96	N/A	Roche

* Extraction of total RNA was performed from fresh snap-frozen tissue samples (~30 mg) following mechanical digestion (GentleMACs M-tubes; program RNA 01_01; Miltenyi Biotec, UK) and purification using the QIAGEN RNeasy mini-kit (QIAGEN, Germany) as per the manufacturer's instructions. All samples were treated with DNAse I (RNase-free DNase set, QIAGEN) during extraction. Conversion of mRNA to cDNA was performed using the iScript cDNA synthesis kit (BioRad, USA). Cycling conditions for qRT-PCR: 95°C for 10 seconds, 59°C for 30 seconds and 72°C for 1 second.

Supplementary Table 3: Constituents / protocol of the VAP-1 enzyme assay*

**Reagents**	**Supplier**
Dulbecco's PBS	Sigma
Benzylamine (VAP-1 substrate)	
Semicarbazide (VAP-1 inhibitor)	
Clorgyline	
Pargyline	
Horseradish peroxidase	
Amplex-UltraRed	Life Technologies
**Buffers**	
Coating buffer	8.4 g $NaHCO_3$ + 3.56 g Na_2CO_3 + ddH_2O (to 1 L, *p*H 9.5)
Wash buffer	PBS + 0.05% Tween20
Blocking solution	PBS + 1% BSA
Sample and standard diluent	Dulbecco's PBS + 0.2% Triton X-100
**Kinetic reading protocol ***	
Excitation: 530nm (slit 25)	
Emission: 590nm (slit 35)	
Sensitivity: 20	
Shaking intensity 3; duration 2, before reading	
Kinetic reading: 10 readings/well; followed over 1 hour	

Liver/colonic protein lysates (60–100mg) were prepared from explanted or resected tissue in lysis buffer (Dulbecco's PBS+0.2% Triton X-100; Sigma) [35] and the concentrations measured using the bicinchoninic acid assay (Pierce Thermo Scientific, UK). Prior to commencing the enzyme assay, all protein concentrations were standardised to 2mg/mL. Kinetic readings per-sample were obtained via rate of change of fluorescence as measured by a Synergy HT Multi-Mode Microplate reader (BioTek) and the enzyme activity calculated from the slope of a standard curve (standard concentrations of H_2O_2 0.2–3.0mM).

Abbreviations: BSA, bovine serum albumin; PBS, phosphate-buffered saline; VAP, vascular adhesion protein.

Supplementary Table 4: Antibodies used for flow cytometry and cell sorting

**Conjugated antibodies (supplier)**	**Isotype (clone)**	**Stock Conc.**	**Working vol. (µl / 10^5 cells)**
Mouse anti-CD3 PE-Cy7 (BioLegend)	IgG1 (SK7)	0.2 mg/mL	5
Mouse anti-α4 integrin APC (BD Biosciences)	IgG1 (9F10)	0.2 mg/mL	2
Rat anti-β7 integrin PE (BD Pharmingen)	IgG2a (FIB504)	0.2 mg/mL	10
**Isotype-matched control (IMC)**			
Mouse PE-Cy7 (BD Pharmingen)	IgG1 (MOPC-21)	0.2 mg/mL	5
Mouse APC (BD Pharmingen)	IgG1 (MOPC-21)	0.2 mg/mL	5
Rat PE (BD Pharmingen)	IgG2a (R35-95)	0.2 mg/mL	10

Supplementary Table 5: sVAP-1 ECTRF assay

**A) Reagents and preparation**	
**Reagents**	
Wash solution	DELFIA® wash concentrate (PerkinElmer, USA)
Sample buffer	DELFIA® assay buffer (PerkinElmer)
Enhancement solution	DELFIA® enhancement solution (PerkinElmer)
Capture antibody	Biotinylated anti-VAP-1 antibody (TK8-14, mouse anti-human IgG2a) (Biotie).
Tracer antibody	Europium coupled anti-VAP-1 antibody (TK8-18, mouse anti-human, IgG2a) (BioTie)
Positive control	Purified soluble human sVAP-1 (Biovian)
**Instruments and materials**	
Victor^3-1420-012- microplate reader + WorkOut 2.5 software (PerkinElmer)	
1296-026 DELFIA® Plate washer (PerkinElmer)	
1296-003 DELFIA® Plate shaker (PerkinElmer)	
1296-041 DELFIA® Plate Dispenser (PerkinElmer)	
4009-0010 DELFIA® Streptavidin 96-well plates (PerkinElmer)	
**Preparation**	
Wash solution	DELFIA® wash concentrate diluted 1:25 in ddH_2O.
Sample dilution	Study samples diluted 1:10
Calibration standards	sVAP-1 reference material + sample buffer
QC samples	4.5 ng/mL (low), 50 ng/mL (medium) and 1200 ng/mL (high)
Capture antibody	Diluted in sample buffer (3 µg/mL) and sterile filtered
Tracer antibody	Diluted in sample buffer (2 µg/mL) and sterile filtered

**B) Assay protocol**
10 µL calibration standards, QC samples and test serum (diluted) added per well
50 µL biotin-europium antibody solution added immediately to each well.
ELISA plate sealed with tape and incubated on plate shaker (2 hours, ambient temperature)
ELISA plate washed x 4 with wash solution
200 µL DELFIA® enhancement solution added to each well and incubated on plate shaker (min. 5 minutes – max. 60 minutes, ambient temperature)
Time-resolved fluorescence measured at 615 nm

Levels were measured by time-resolved fluorescence at 615 nm (Victor^3 multi-label counter, PerkinElmer) by comparing to a reference sample (positive control) of highly purified human sVAP-1 (Biovian, Finland). The assay calibration ranged from 1.5 – 1500ng/mL and linearity (R^2) of calibration curves 0.997 – 1.000. A nonlinear, weighted (1/x) 4-parameter logistic model was used to fit the calibration curve ([y = a + (d – a) / (1 + (x / c) ^b), weighting 1/x; where c is the centre point (ED50), d is the upper limit, a is the lower limit, b is the relative slope around c]).
Abbreviations: ECTRF, Europium-coupled time-resolved fluorescence; ELISA, enzyme-linked immunosorbent assay; QC, quality control; sVAP; soluble vascular adhesion protein.

Supplementary Table 6: VAP-1 substrate kinetic profiling

**Substrate**	**V_{max}^{app} Mean + SEM**	**K_m^{app} (μM) Mean + SEM**	**k_{cat}^{app} (s^{-1})**	**k_{cat}^{app}/K_m^{app}**
Ethylamine	8.59 ± 0.60	88.44 ± 34.90	242.63	2.74 x 10^6
Phenethylamine	9.89 ± 0.69	65.69 ± 19.43	279.46	4.25 x 10^6
Benzylamine	8.92 ± 0.70	9.90 ± 3.360	251.98	2.55 x 10^7
Methylamine	11.49 ± 0.77	8.55 ± 3.45	324.58	3.80 x 10^7
Dopamine	78.94 ± 5.97	43.70 ± 13.07	2229.94	5.10 x 10^7
Cysteamine	84.25 ± 5.64	43.98 ± 16.74	2379.69	5.36 x 10^7

Substrate kinetic profiles were estimated from the Michaelis Menten equation $v' = V_{max}^{app}$ [S]/K_m^{app}+[S] where $V_{max}^{app}=k_{cat}^{app}[E]_o$. The higher the ratio between k_{cat}^{app}/K_m^{app}, the greater the enzymatic efficiency. All reactions were carried out at 37°C and 21% O_2.

Supplementary Table 7: Comparison of final PSC derivation and validation cohorts comprising clinical outcome studies

**Characteristic**	**Derivation** (*n* = 104)	**Validation** (n = 80)	***p* value**
Patient age *	42yrs. (28 – 58) yrs.	40 (31 – 53) yrs.	0.427
Male gender	65 (63)	65 (81)	0.006
IBD	81 (78)	58 (73)	0.489
Colonic IBD	81	58	
Unknown distribution	-	-	
History of colonic dysplasia/cancer	5 (5)	5 (6)	0.749
Immunosuppression	18 (17)	16 (20)	0.703
Steroids	4	16	
Anti-purinergic	6	0	
Steroids + anti-purinergic	9	0	
Calcineurin inhibitor	7	0	
Anti-TNFα	1	0	
UDCA exposure *	70 (67)	34 (43)	< 0.001
Laboratory parameters *			
Serum AST (ratio to ULN)	1.58 (0.85 – 2.40)	1.72 (1.13 – 3.34)	0.258
Serum ALT (ratio to ULN)	1.48 (0.85 – 2.60)	1.10 (0.60 – 2.00)	0.080
Serum ALP (ratio to ULN)	2.03 (0.94 – 3.22)	2.30 (1.50 – 3.40)	0.076
Bilirubin	18 (10 – 33)	20 (12 – 39)	0.225
Albumin	43 (39 – 45)	41 (36 – 43)	0.001
Platelet count	215 (136 – 297)	290 (217 – 375)	<0.001
INR	1.0 (1.0 – 1.1)	1.0 (1.0 – 1.0)	<0.001
Sodium	141 (139 – 143)	140 (139 – 142)	0.055
Creatinine	72 (61 – 84)	66 (57 – 73)	0.004
pANCA positive	40 (37)	34 (35)	0.995
Serum VAP-1 level ng/mL (immunofluorescence method) *	533 (428 – 663)	-	-
Serum VAP-1 level ng/mL (chemiluminescence method) *	928 (693 – 1187)	751 (644 – 991)	0.010
MELD score *	6 (6 – 8)	6 (6 – 8)	0.277
Clinical events, *n* (incidence rate)	31 (11.3 events / 100-pt.yrs.)***	25 (19.5 events / 100-pt.-yrs.)***	0.124
LTx	24	16	
Death	10	11	
Follow-up interval duration **	41 (31 – 51) months	44 (33 – 56) months	0.977

Categorical variables expressed as number (percentages). Continuous variables expressed as median (interquartile ranges).
* At time of sampling. Paired laboratory data unavailable in 24 patients from the validation cohort.
** Median follow-up duration (IQR) determined from point of sampling till clinical event (liver transplantation or death).
*** Calculated as incidence rate to first clinical event in patients experiencing both liver transplantation and death.
Abbreviations: ALT, alanine transaminase; ALP, alkaline phosphatase; AST, aspartate transaminase; IBD, inflammatory bowel disease; INR, international normalised ratio; MELD, model for end-stage liver disease score. pANCA, peri-nuclear anti-neutrophil cytoplasmic antibody; TNF, tumour-necrosis factor; UDCA, ursodeoxycholic acid.

Supplementary Figure 1: Michaelis-Menten kinetic modelling of substrate-induced VAP-1 activity

Substrate kinetic profiles were calculated as indicated by the equation provided; wherein the reaction rate increases with increasing substrate concentration, asymptotically approaching its maximum rate $V_{\text{max,}}$ attained when all enzyme is bound to substrate. The enzymatic efficiency is calculated as: k_{Cat} / K_m.

Supplementary Figure 2: Vascular adhesion protein (VAP)-1 expression in non-diseased and PSC liver

Immunohistochemical detection of VAP-1 (red) in non-diseased and explanted PSC liver. Representative images of *n*=7 are shown [A] for sinusoidal staining in non-diseased liver, with higher magnification of a centrilobular region [B] and portal tract [C]. PSC liver sinusoidal staining is shown in [D], a fibrous septum in [E], and a portal tract in [F]. Hepatic vein [h], portal vein [p] and hepatic artery [a]. Scale bars: 100 μm.

Supplementary Figure 3: Sinusoidal vascular adhesion protein (VAP)-1 expression in autoimmune liver disease

Immunohistochemical detection of VAP-1 was conducted in patients with primary sclerosing cholangitis (PSC; *n*=7), primary biliary cholangitis (PBC; *n*=5), autoimmune hepatitis (AIH; *n*=5), and non-diseased liver (NL; *n*=7). Representative images showing differences in sinusoidal staining intensity are provided (ImmPACT^{TM} DAB as the chromogen), together with respective isotype-matched control (IMC) sections. Scale bars: 100 μm.

Supplementary Figure 4: Confocal immunofluorescence of hepatic VAP-1 expression and αSMA in PSC

Confocal immunofluorescence microscopy was performed in PSC liver, to show colocalisation of VAP-1 with αSMA in portal and septal vessels, as well as with stromal staining in fibrotic septa. Representative images from *n*=5 cases are shown, with VAP-1 in green and αSMA in red. Scale bars: 100 μm.

Supplementary Figure 5: Confocal immunofluorescence of hepatic VAP-1 expression and CD31 in PSC

Confocal immunofluorescence microscopy in PSC liver sections showing the proximity of VAP-1 to $CD31^+$ endothelial staining in portal and septal vessels. Representative images from *n*=5 cases are shown, with VAP-1 in green and CD31 in red. Scale bars 100 μm.

Supplementary Figure 6: VAP-1 does not co-localise with biliary epithelium

Co-expression of VAP-1 in green was investigated with the biliary marker epithelial cell adhesion molecule (EpCAM) shown in red via multi-colour confocal immunofluorescence in PSC liver. Representative images from *n*=5 cases are shown Scale bars: 100 μm.

Supplementary Figure 7: VAP-1 mRNA expression in human liver

Absolute quantification of VAP-1 gene expression in explanted liver tissue is presented for autoimmune hepatitis (AIH; *n* = 5), primary biliary cholangitis (PBC; *n* = 5), primary sclerosing cholangitis (PSC; *n* = 7) and non-diseased resected liver tissue (NL; *n* = 7).

Supplementary Figure 8: Enrichment of $\alpha 4\beta 7^+$ T-cells from peripheral blood

Fluorescence-activated cell (FAC)-sorting was utilised to enrich the proportion of circulating $\alpha 4\beta 7^+$ T-cells from blood donors to over 90% purity

Supplementary Figure 9: Accuracy of sVAP-1 Levels in Predicting Clinical Outcomes in PSC

An ROC curve illustrating the predictive value of sVAP-1 in predicting clinical outcome is presented for [A] the immunofluoresecence assay (AUROC: 0.732, 95% C.I.: 0.608 – 0.857; p <0.001); and [B] the chemiluminescence assay (AUROC: 0.740; 0.626–0.848, p<0.001 Patients listed for transplantation / < 3 months' follow-up at time of serum sampling were excluded from the analysis to avoid bias. Abbreviations: AUROC, area under the ROC curve; ROC, receiver operator characteristic; C.I., confidence interval.

Supplementary Figure 10: Predictive Value of Elevated sVAP-1 in PSC Patients with a Normal Bilirubin, Albumin and Platelet Count

Kaplan–Meier survivorship estimates indicate the progression of primary sclerosing cholangitis (PSC) in the Birmingham derivation cohort, taken from the time of serum sampling in specific patient subgroups; specifically those with [A] a normal serum bilirubin (<21 μmol/L), [B] a normal serum albumin (>35 g/L), [C] a normal circulating platelet count (>150 x 10^9/L), and [D] normal levels of all three parameters. Disease progression stratified according circulating sVAP-1 values (immunofluorescence method) is shown according to an AUROC-derived cut-point of 529 ng/mL. Patients with an sVAP-1 below this cut-point represent group A and those with a value above fall into group B. Clinical events are indicated as liver transplantation or liver-related death. Hazard ratios (HR) for group B vs. group A in each panel are indicated in parenthesis.

Supplementary Figure 11: Correlation between serum sVAP-1 measurements between assays.

Serum sVAP-1 levels were measured in 134 patients with PSC. Method 1 was conducted at the Biotie Corp. laboratory and used an immunofluorescence technique. These results were correlated with those derived from the University of Turku, which applied chemiluminescence detection, method 2.

Supplementary Figure 12: Clinical outcomes in derivation and validation cohorts stratified by circulating sVAP-1 values

Kaplan-Meier estimates of liver transplant free survival for both derivation (Birmingham, UK) and validation cohorts (Oslo, Norway) are shown in [A]. Disease progression was then stratified according to circulating sVAP-1 levels (chemiluminescence method), determined via an AUROC-derived cut-point of 923 ng/mL in the derivation cohort. Patients with an sVAP-1 level below this cut-point represent group A' and those with a value above fall into group B'. Survival curves are shown for both cohorts combined in [B]; the derivation cohort only [C]; and the validation cohort only [D].

Supplementary Figure 1

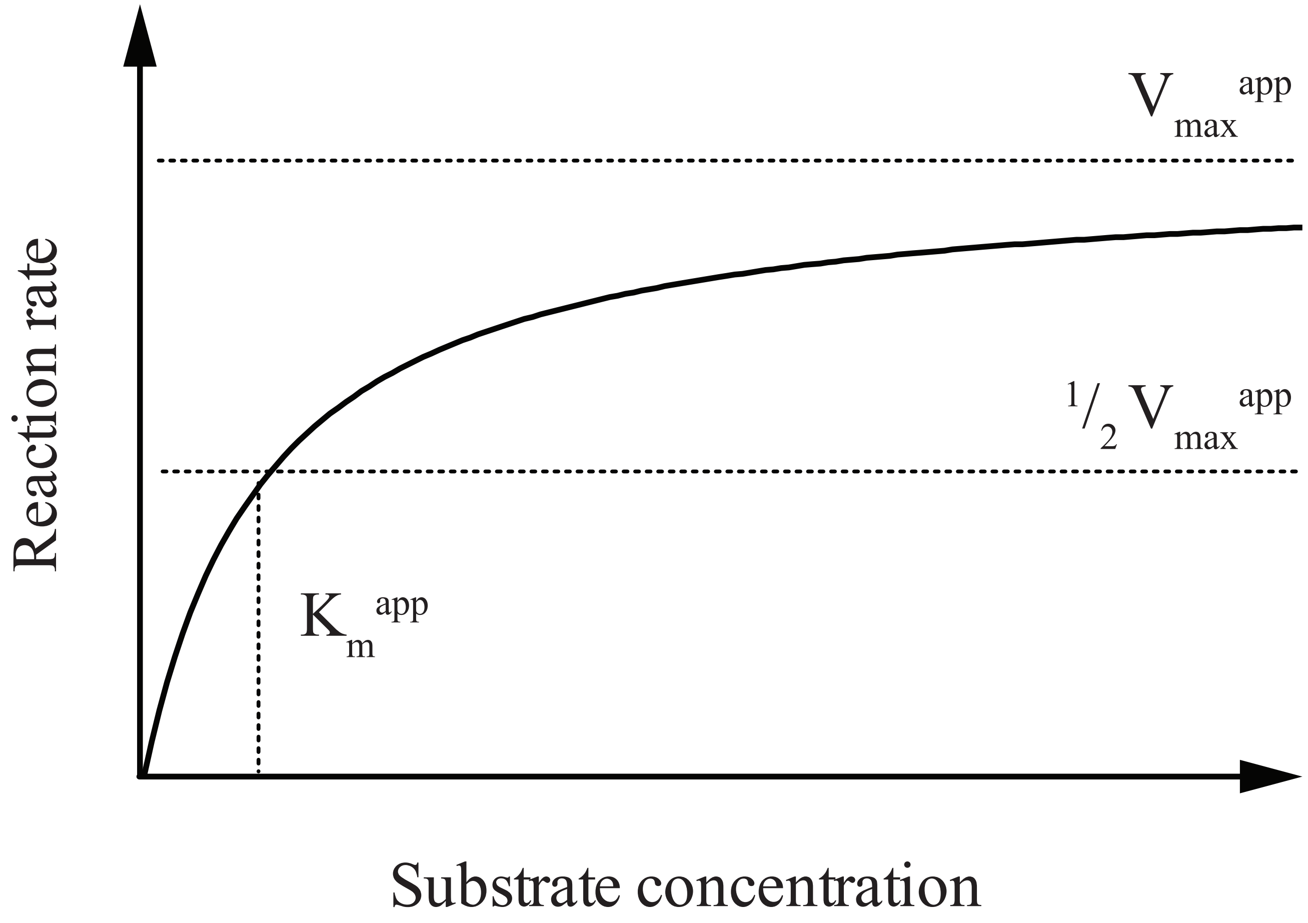

Equation:

$v' = V_{max}^{app}$ [S] / K_m^{app} + [S].

Wherein: $V_{max}^{app} = k_{cat}^{app}$ [E] o

Supplementary Figure 2

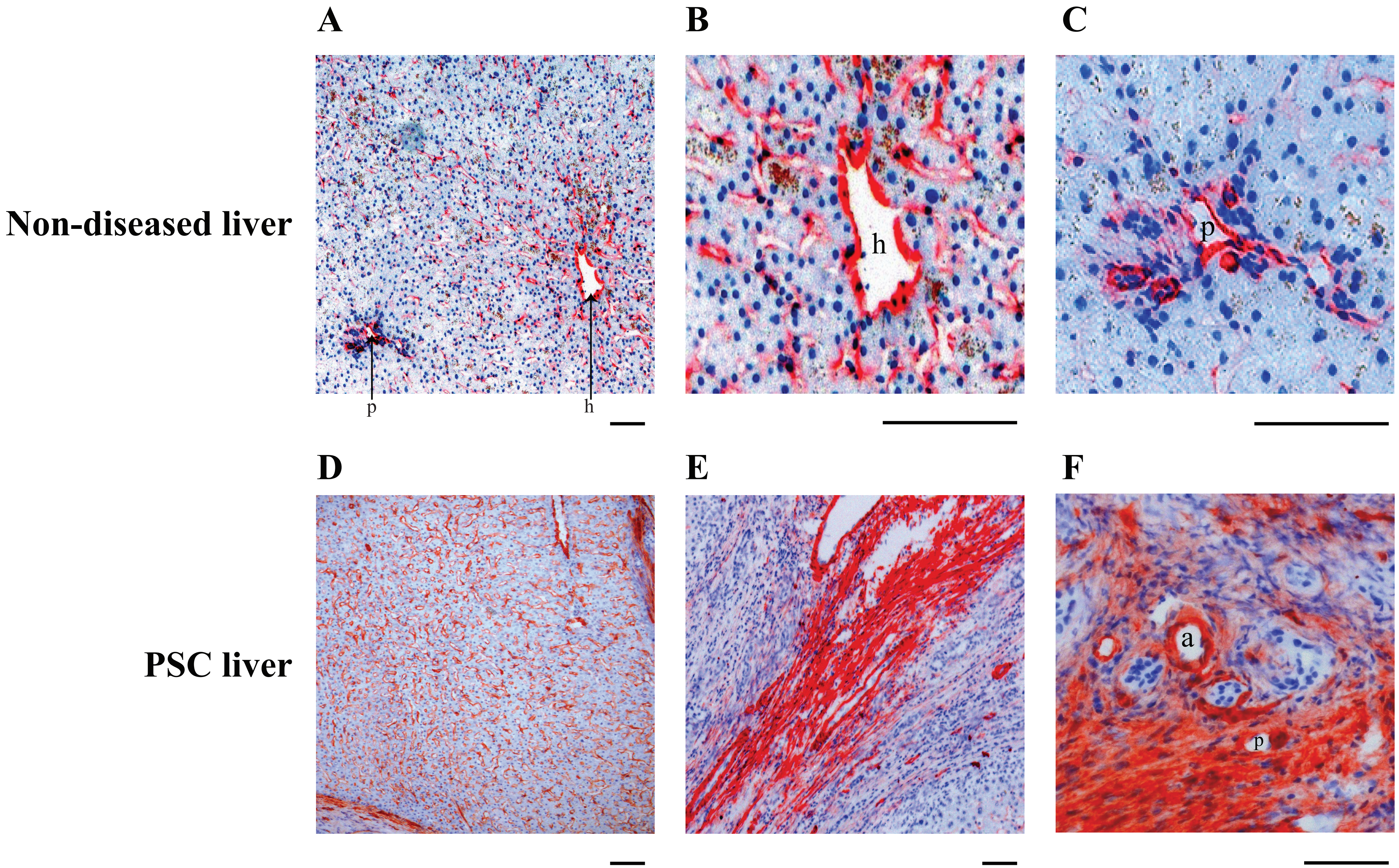

Supplementary Figure 3

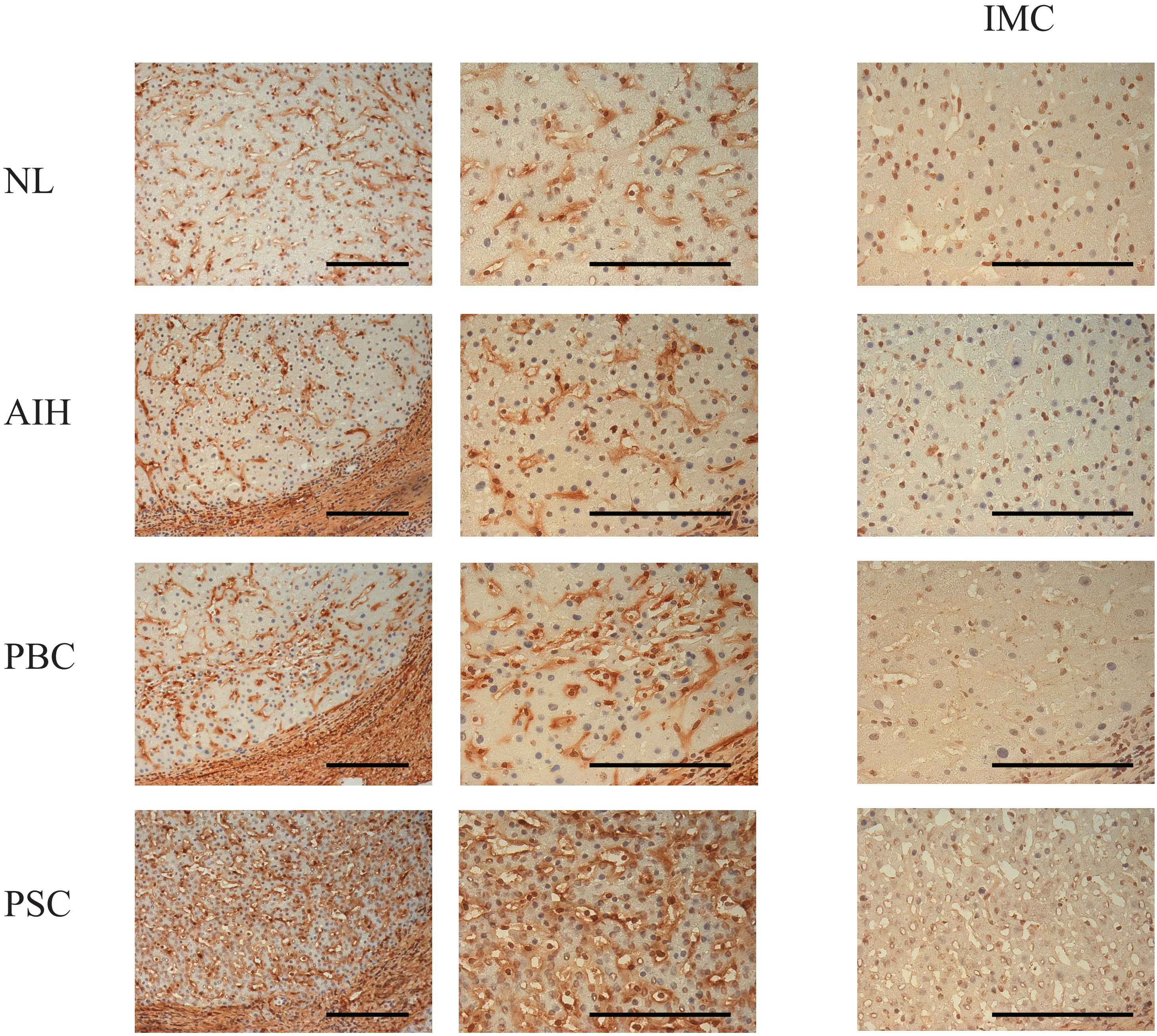

Supplementary Figure 4

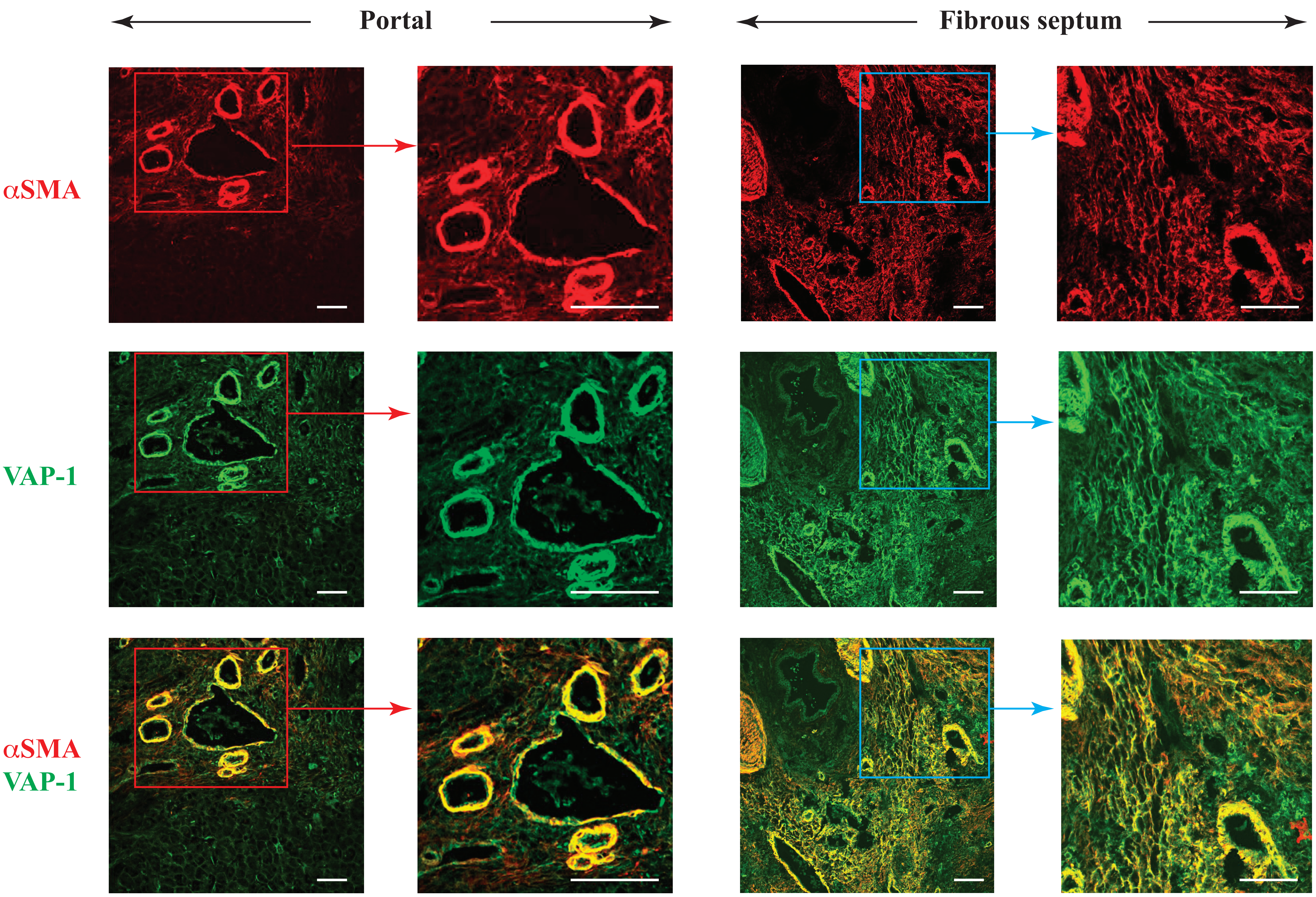

Supplementary Figure 5

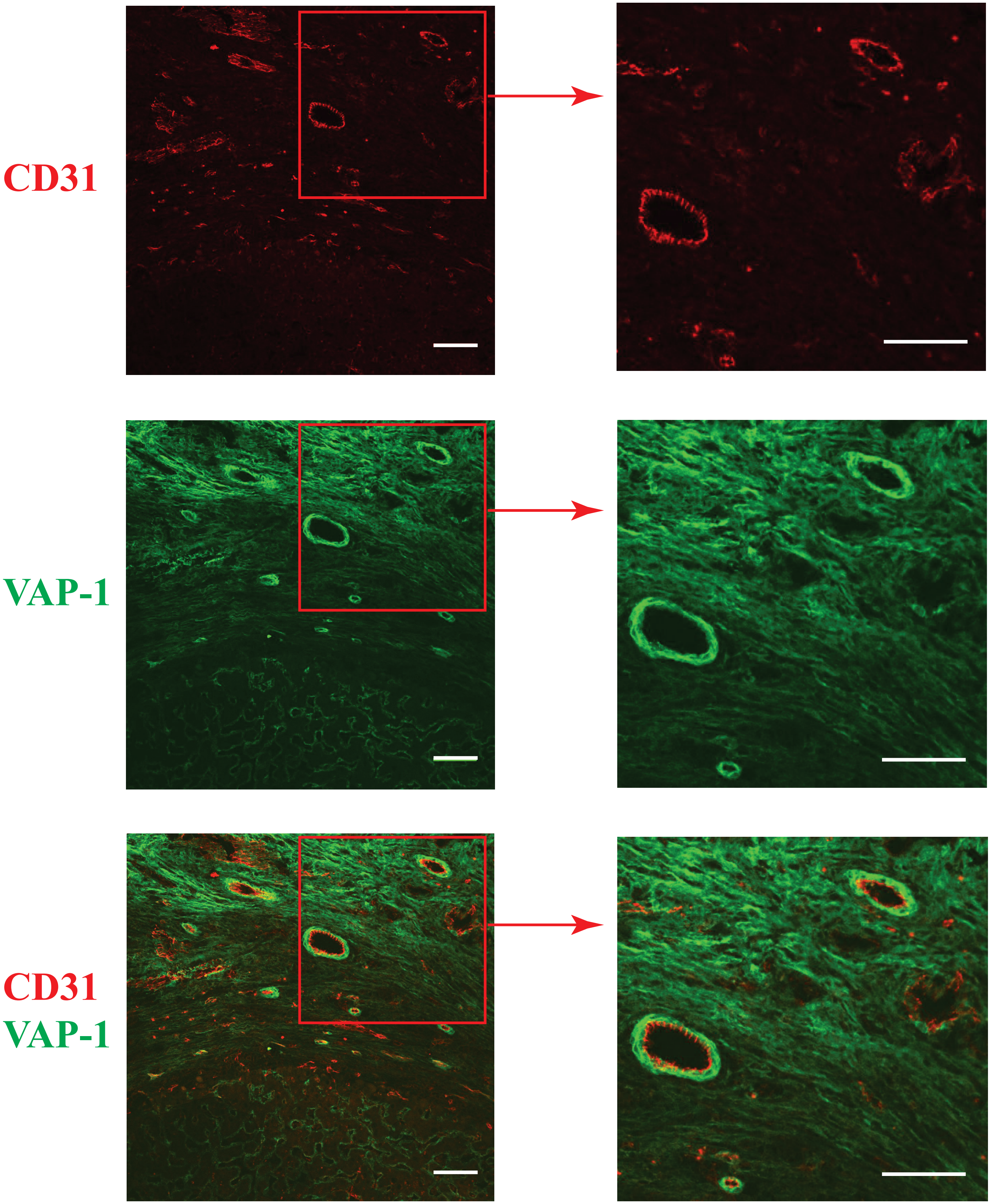

Supplementary Figure 6

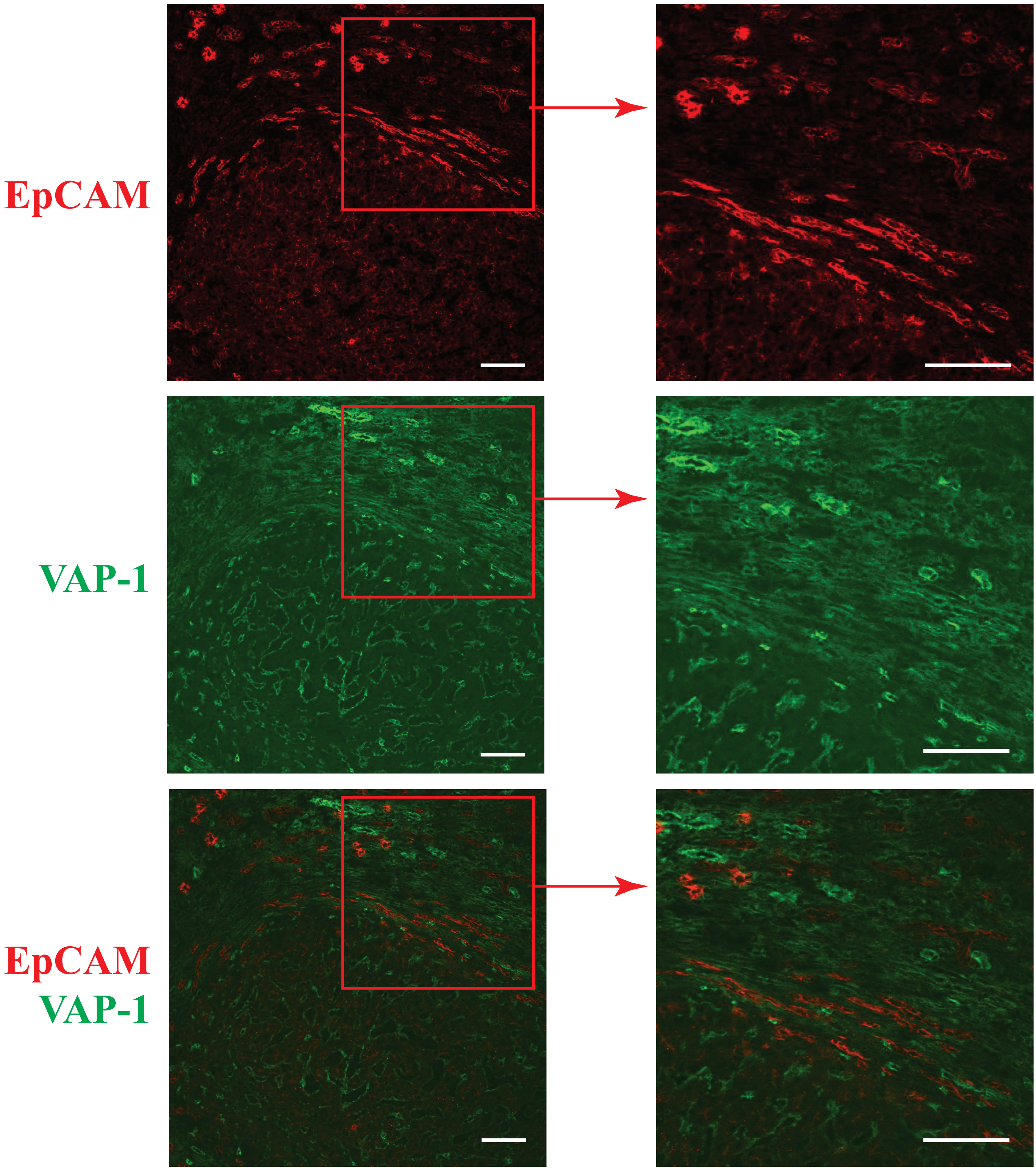

Supplementary Figure 7

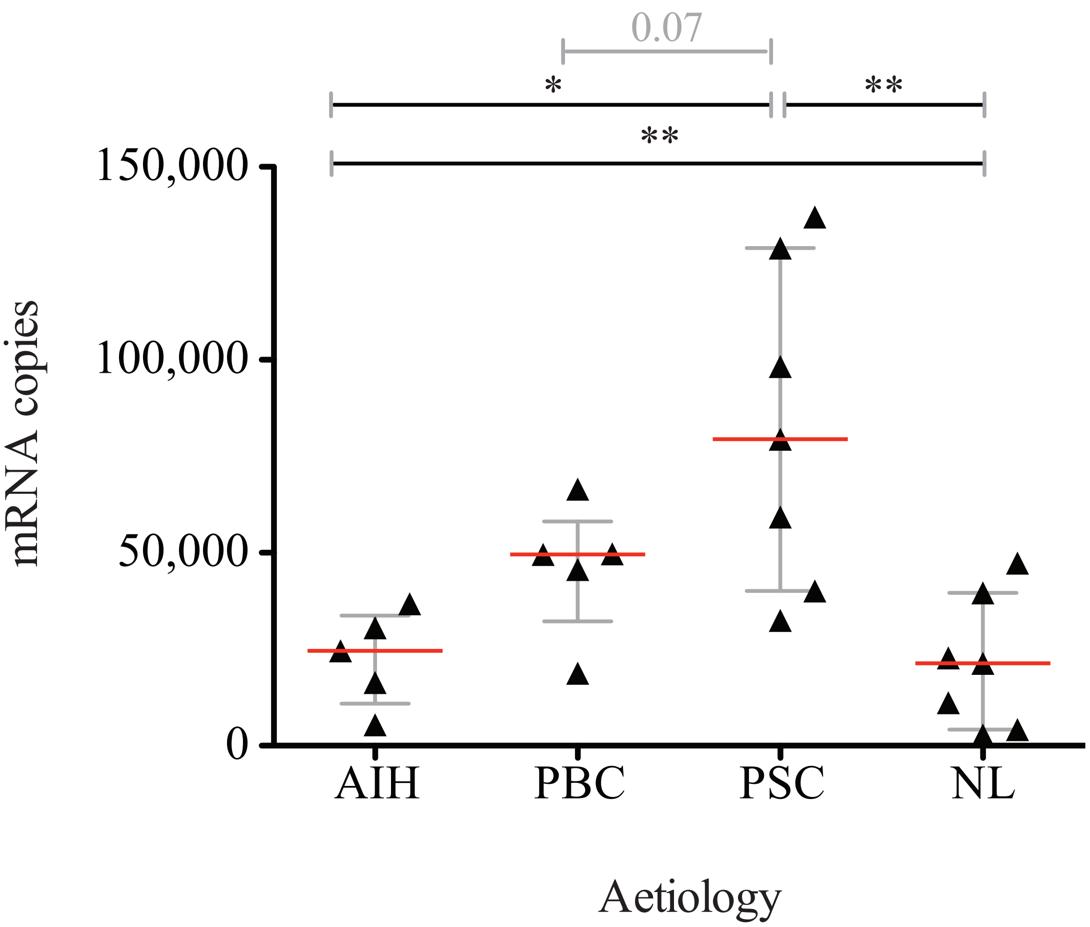

* p < 0.05
** p < 0.01

Supplementary Figure 8

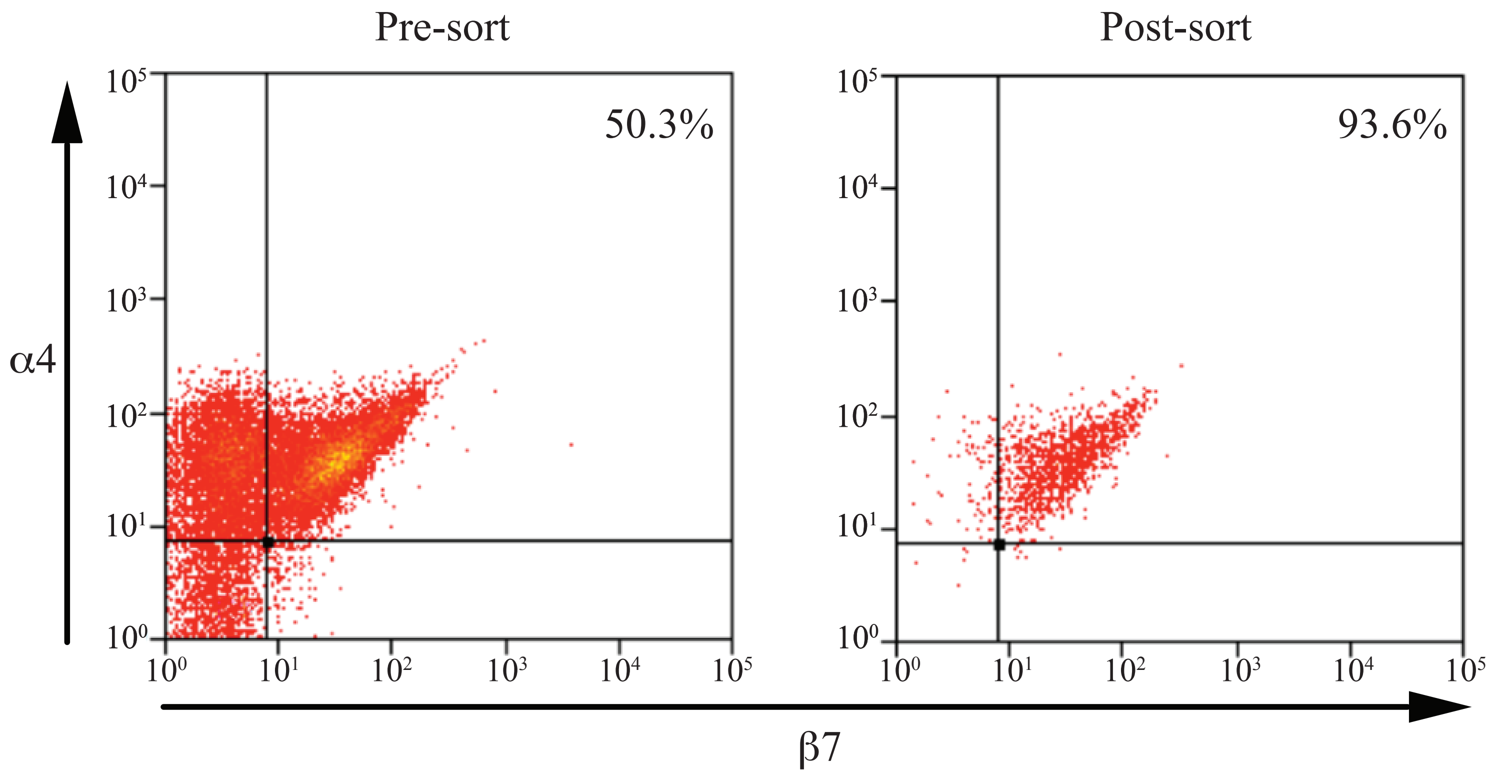

Supplementary Figure 9

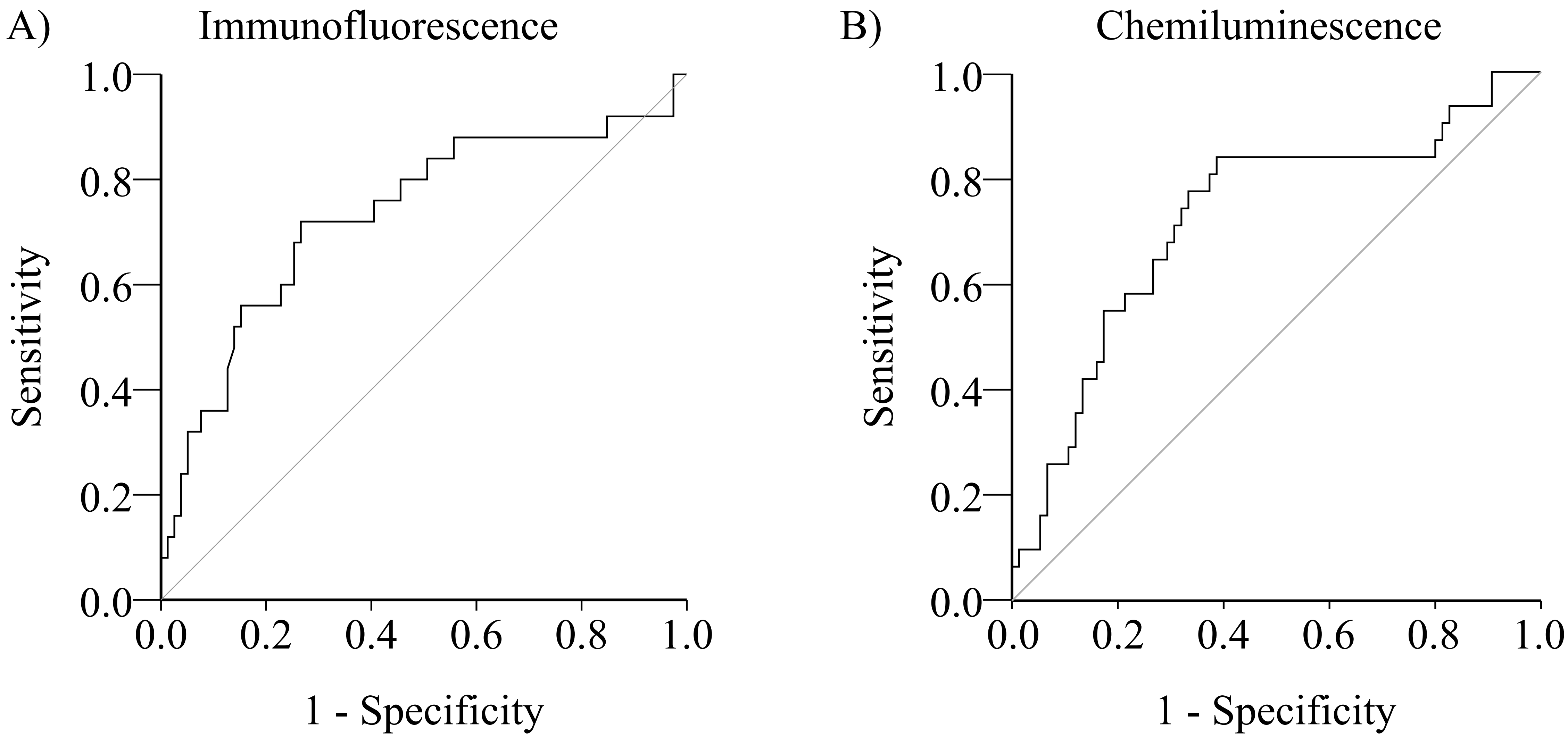

Supplementary Figure 10

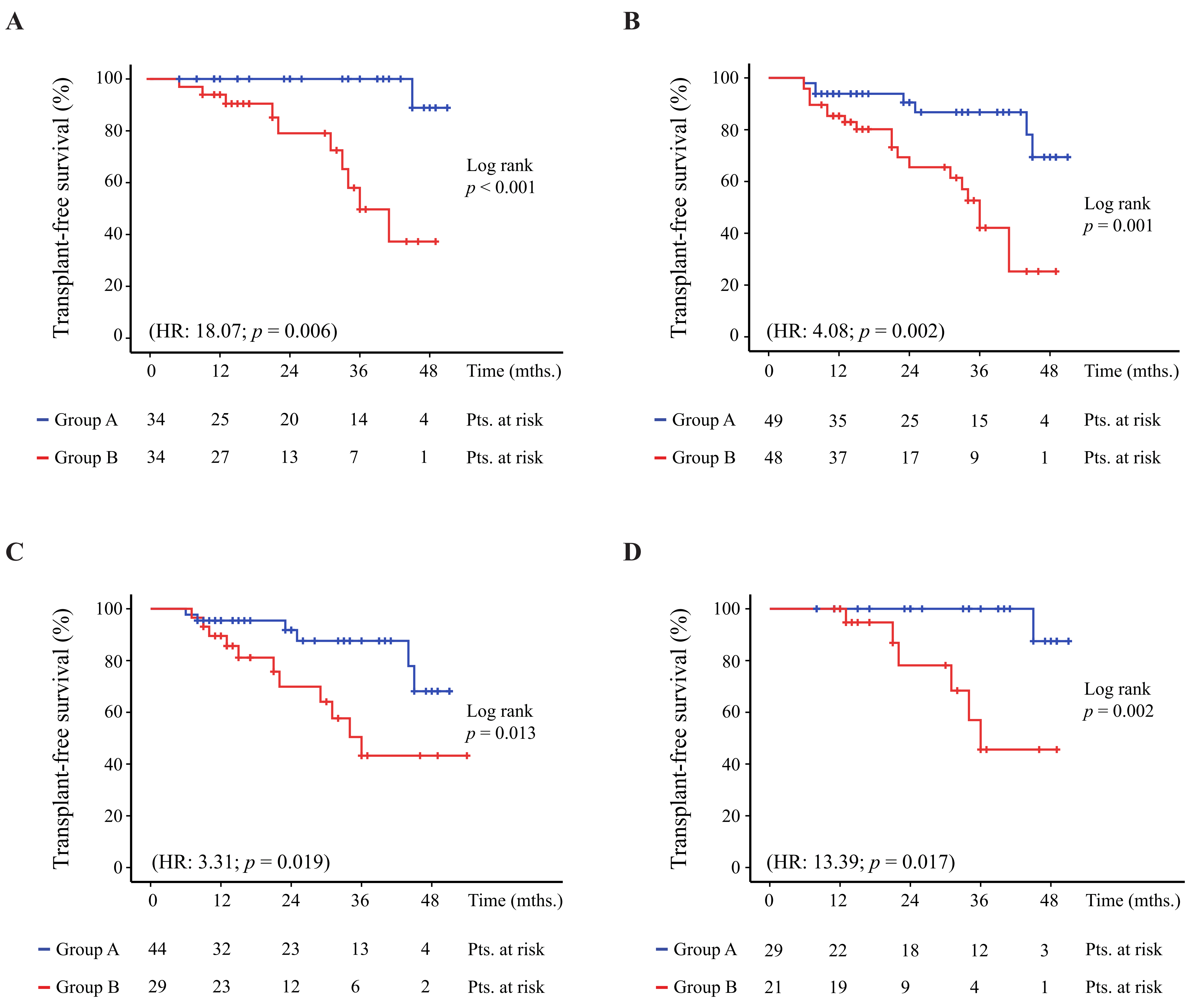

Supplementary Figure 11

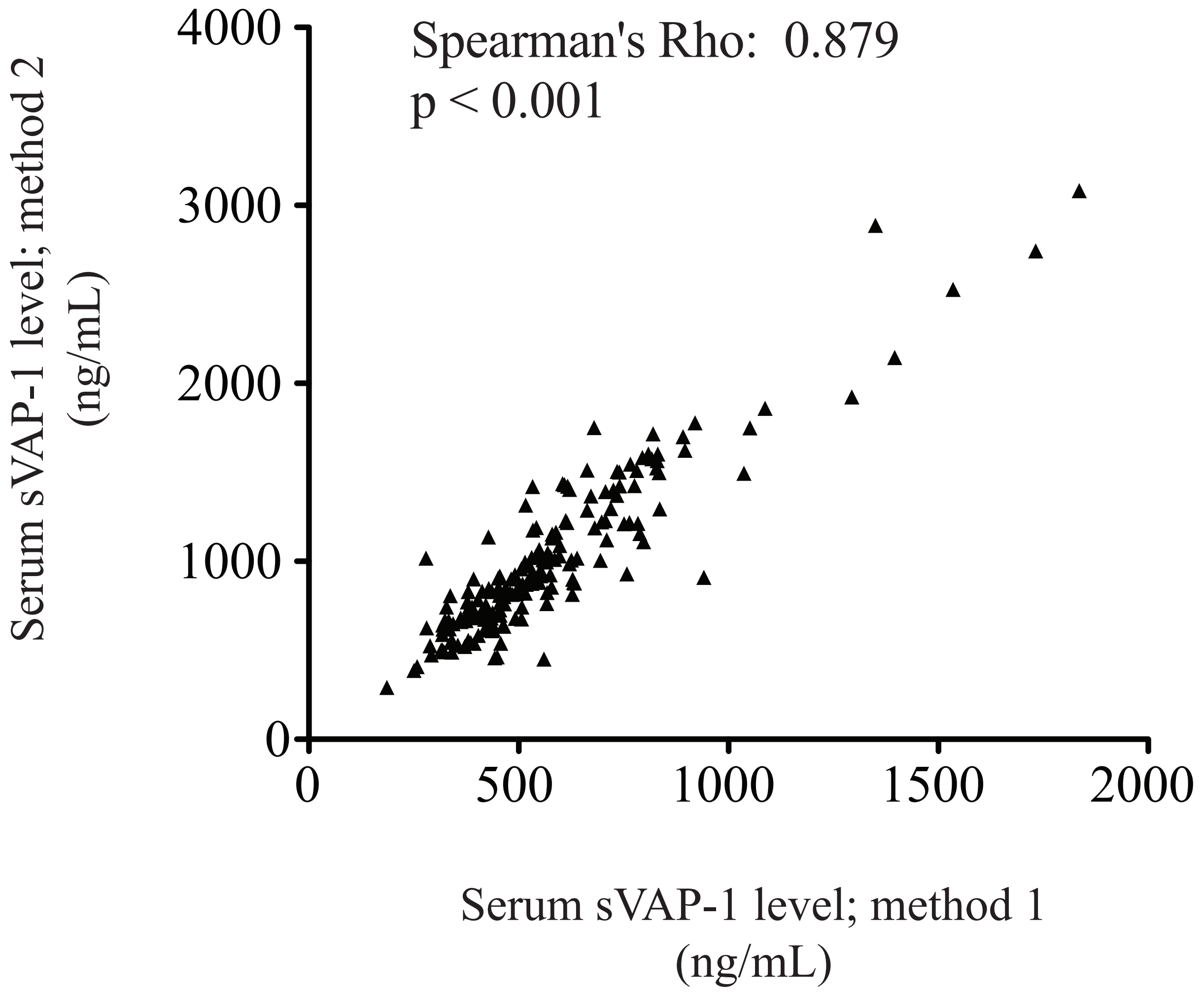

Supplementary Figure 12

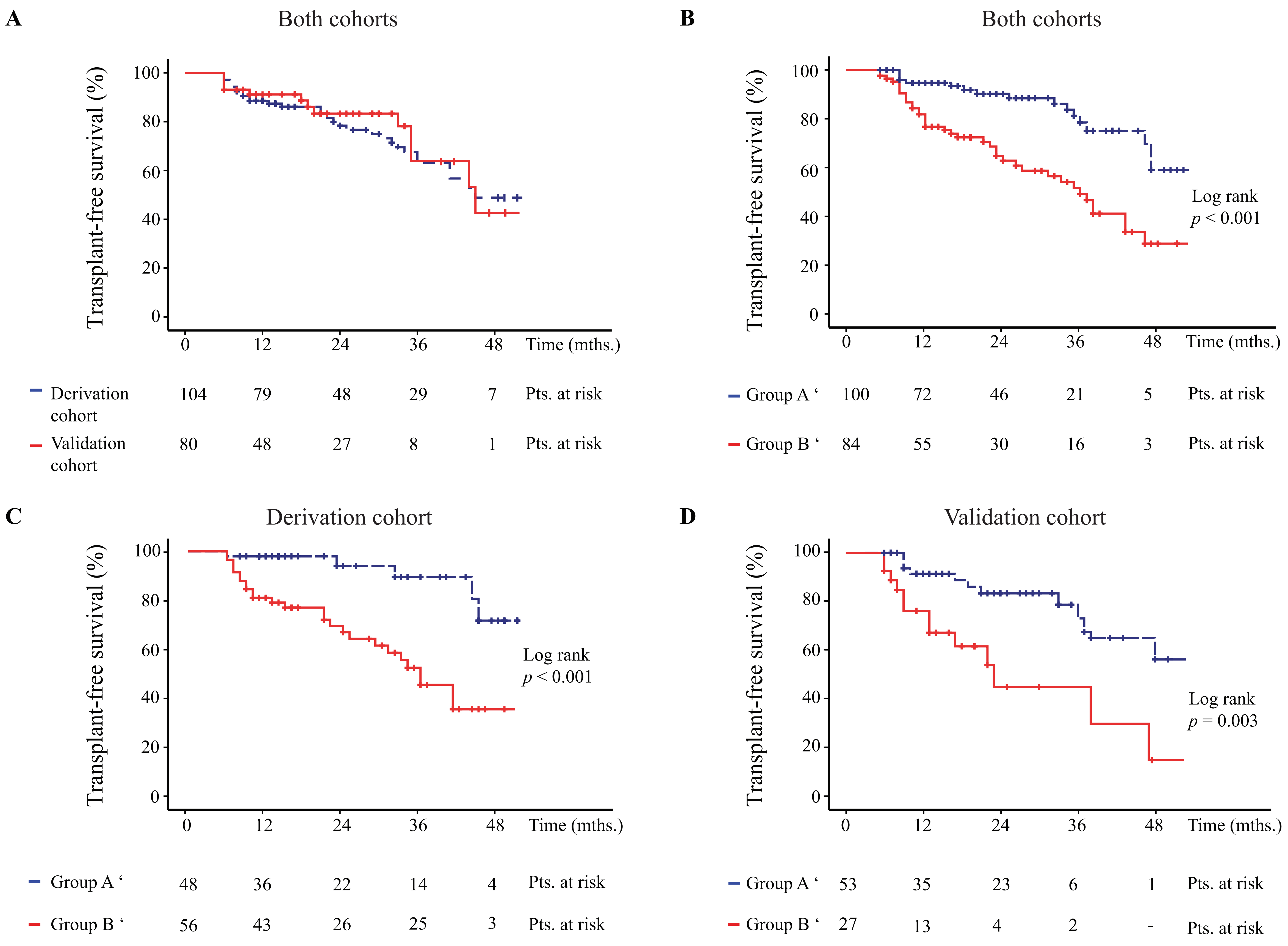

Supplement: supplementary data [file gutjnl-2016-312354supp001.pdf]
